# Dynamical persistence in high-diversity resource-consumer communities

**DOI:** 10.1371/journal.pcbi.1008189

**Published:** 2020-10-12

**Authors:** Itay Dalmedigos, Guy Bunin

**Affiliations:** Department of Physics, Technion-Israel Institute of Technology, Haifa 32000, Israel; University of Illinois at Urbana-Champaign, UNITED STATES

## Abstract

We show how highly-diverse ecological communities may display persistent abundance fluctuations, when interacting through resource competition and subjected to migration from a species pool. These fluctuations appear, robustly and predictably, in certain regimes of parameter space. Their origin is closely tied to the ratio of realized species diversity to the number of resources. This ratio is set by competition, through the balance between species being pushed out and invading. When this ratio is smaller than one, dynamics will reach stable equilibria. When this ratio is larger than one, the competitive exclusion principle dictates that fixed-points are either unstable or marginally stable. If they are unstable, the system is repelled from fixed points, and abundances forever fluctuate. While marginally-stable fixed points are in principle allowed and predicted by some models, they become structurally unstable at high diversity. This means that even small changes to the model, such as non-linearities in how resources combine to generate species’ growth, will result in persistent abundance fluctuations.

## Author summary

Competition over resources is one of the main mechanisms by which species interact. In recent years, considerable interest has been directed towards species-rich communities that compete over resources. An important and basic question concerns the dynamics of such communities: do abundances reach stable equilibria, or continue to fluctuate even without environmental fluctuations? Much of the work on high-diversity resource-competition models starts from the highly-influential MacArthur model or similar models, known to always relax to equilibria at long times, seemingly precluding any long-time non-equilibrium phenomena. And so while long-lived chaos has been demonstrated in few-species models, the question of persistent dynamics at high-diversity remains open. We show that high-diversity resource-competition models generically have a regime of parameters in which abundances do not reach equilibria. The MacArthur model and related models, which always relax to equilibrium, are in fact structurally unstable in that regime: when small changes are made to the model, non-equilibrium dynamics appear. We present a theoretical framework: we identify the relevant model parameters, and explain when long-time abundance fluctuations appear in a predictable and reproducible way. We show that diversity may significantly exceed the bound set by competitive exclusion.

## Introduction

Resource competition is one of the main mechanisms underlying species interactions. Theoretical works [1, 2] have demonstrated that communities interacting via resource competition may exhibit different dynamical behaviors (also observed in nature [3, 4]), including relaxation to equilibria, limit cycles and chaotic dynamics. In systems consisting of a few species and resources, the dynamical outcome may depend on all the details describing the interactions in the community [5]. For systems with higher dimensionality (more species and resources), full detailed knowledge of the interactions may be difficult to obtain and predictions might seem hopeless, and potentially sensitive to all unknown details.

In recent years, research on high-dimensional communities has shown that full knowledge on all the interactions might not always be needed [6–14], and important ecological quantities such as total biomass and diversity can be predicted from a handful of statistics on the interaction parameters. In the space of these relevant statistics, one can identify different regions (referred to as “phases”) with qualitatively distinct behaviors, such as relaxation to equilibria versus chaotic dynamics. Within these phases, the qualitative behavior is robust, i.e. insensitive to sufficiently small changes in the systems’ interaction coefficients.

In this paper, we consider high-dimensional communities with resource-competition interactions. We show that in an entire region of parameter space, the system fails to reach equilibria and instead abundances fluctuate indefinitely. This might seem surprising, as some theoretical models are known to always lead to stable equilibria, including classical models by MacArthur [15, 16]. We argue that when the number of species and resources is large, there are regions of parameter space where these models are highly sensitive (structurally unstable), and even very small changes to the model will result in persistent abundance fluctuations.

A key ingredient in our discussion is competitive exclusion, according to which the number of species that can coexist in a stable equilibrium is smaller or equal to the number of resources (or more generally, the number of niches). A *marginally*-stable fixed point, on the border between stability and instability, *can* accommodate more species than resources, but it can be destroyed by small perturbations or changes to the dynamical rules.

The sensitivity of marginally-stable equilibria raises the following question: what then replaces the marginally stable fixed-point, once it is no longer stable? There are two possible scenarios: (1) Species will go extinct until an equilibrium with fewer species is reached, which satisfies the competitive exclusion principle, or (2) The system will not reach any fixed point, and instead abundances will continue to fluctuate indefinitely. We show that for a community experiencing migration from a species pool, the fate of fixed points close to marginal stability is generically number (2) above.

Resource competition dynamics in diverse communities have been analyzed in a number of works employing tools from statistical physics [7, 10, 13, 17]. For a region of model parameters, marginally-stable [10, 18] or close to marginally-stable [7, 13] equilibria are reached. Yet the models studied all admit a unique equilibrium by construction, either stable or marginally-stable (in the spirit of works by MacArthur [15, 16]). For example, species’ growth rates are assumed to be additive in resource availability, which cannot accommodate effects such as essential resources [19]. A model combining resource-competition with other interactions not mediated by resources, was studied in [7]. It showed that a unique stable equilibrium cannot exist in a certain region of parameter space. We employ a similar setting in one of the models we use, in order to demonstrate the high sensitivity of equilibria in resource competition near competitive exclusion, and we also study what replaces that unique equilibrium.

Communities may be driven towards marginal stability by additional factors, such as metabolic trade-offs [20] or evolution, highlighting the importance of studying the generic dynamics in these situations.

An equilibrium phase and a persistent-dynamics phase are known to exist in high-diversity Lotka-Volterra systems with random interaction strengths [8, 9, 12, 14], which need not derive from underlying resource competition. The present work finds analogous phases in resource-competition dynamics, bringing these two families of models closer than previously thought.

**Fig 1:**
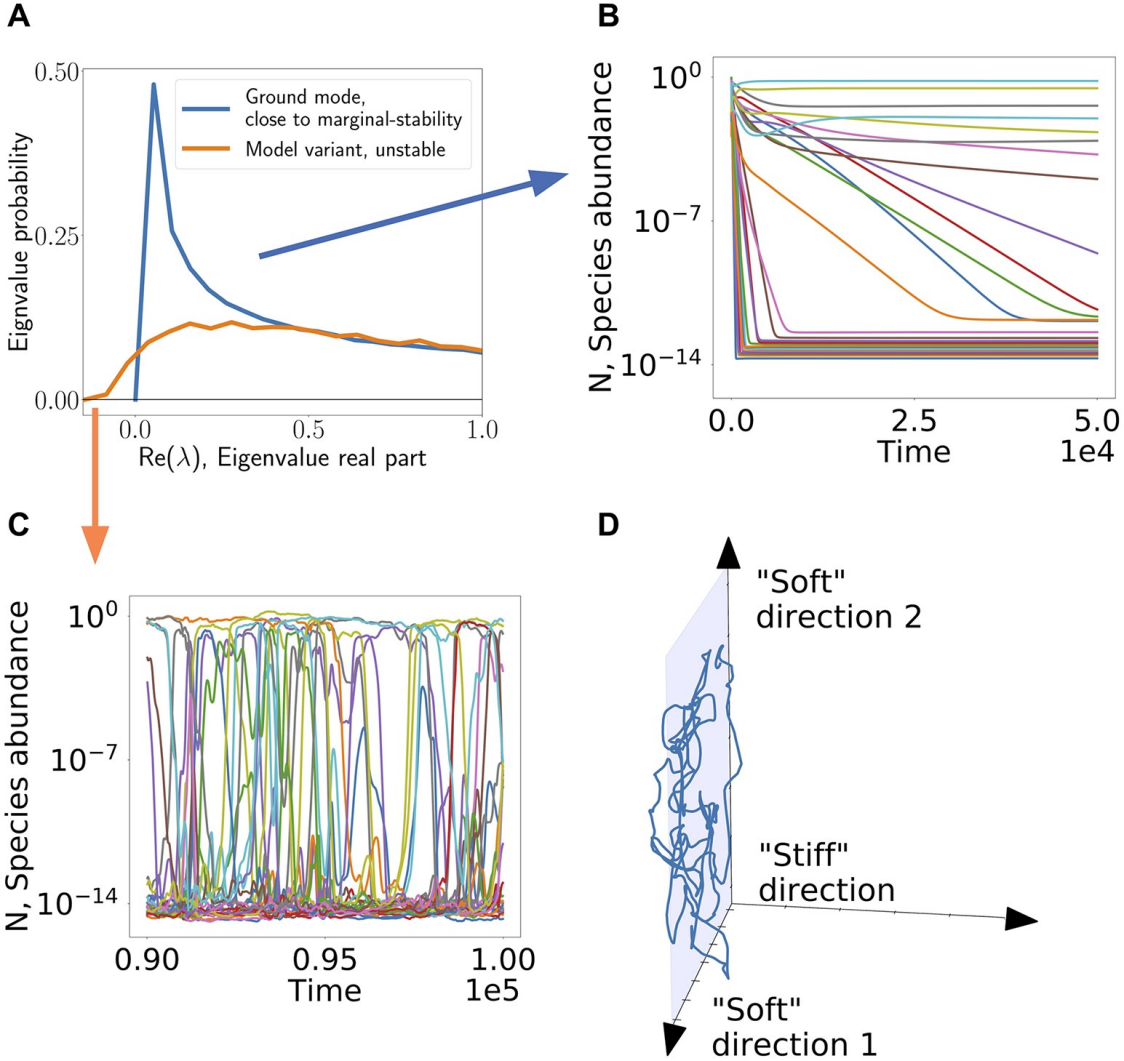
Overview of argument. (A) The spectrum of response to perturbations. Fixed points encountered by a high-diversity resource-competition community may be unstable. (B) Marginally-stable fixed points (or nearly marginal ones) that appear in certain models, are characterized by a non-negative spectrum. The community can relax to a fixed point, but the stability of such fixed points is sensitive to modeling assumptions, including additional interactions of other types, or how growth rates depend on resource availability. (C) In the presence of migration an instability in the fixed point creates persistent abundance fluctuations, in which species are pushed out due to the instability, but are later able to invade again. (D) In such a case, the directions corresponding to the nearly marginal eigenvectors become “soft” directions, showing large fluctuations. For clarity, in (B,C) 30 representative species are plotted.

The following picture emerges, for how persistent dynamics are generated and maintained. Interactions create a balance between species being pushed out due to competition, and species invading when they can, steering the community towards some target species richness. If this richness is larger than the number of resources, then fixed points generically will be unstable, and persist abundance fluctuations will ensue, See Fig 1(C). These dynamics are characterized by species being pushed out by fixed points’ instability, and back when they are able to invade.

This instability is manifested by the spectrum of response to small perturbations around a putative fixed point at the target species richness, Fig 1(A). Under certain modeling assumptions, these fixed points might be marginally stable, but in this case small changes to the model push the fixed point to become truly unstable, without changing much the target richness set by the competition, see Fig 1(C). In other words, it is precisely the large number of (nearly-)marginal directions that allows for such fluctuating dynamics to persist, as shown Fig 1(D). Marginal, or nearly-marginal eigenvectors around the fixed point become “soft” directions, namely combined abundance fluctuations of multiple species that are met with little resistance. This correspondence is further explored in S1 Text (Appendix F).

The paper is structured as follows. Sec. The ground model defines the ground model used to illustrate the arguments. Sec. Demonstrating the sensitivity looks at the effect of changes to the model, by adding interactions on top of resource competition. It shows how the dynamics generated by this model might vary significantly due to even small changes, replacing equilibria by non-equilibrium dynamics. The general mechanism behind this sensitivity is explained in Sec. Theory for the onset of non-equilibrium dynamics. In Sec. Non-linear resource intake, the behavior is shown to be sensitive in a second variant of the model in which all interactions are strictly the result of resource competition, but with non-linear resource intake.

Sec. Species abundance distribution and diversity describes the resulting abundance distributions and community diversity in the dynamically-fluctuating state. The non-equilibrium coexistence of more species than there are resources or niches, is of great interest in its own right. It has been suggested to play a part in the resolution of the “paradox of the plankton” [21]. In Sec. Species abundance distribution and diversity we consider this question directly in a high-dimensional setting, in light of works on high-dimensional chaos in well-mixed communities [6, 14] and meta-communities [22, 23]. Finally, the Conclusions section concludes with a discussion, focusing on predictions for experiments and natural communities.

## Methods and results

### The ground model

To illustrate the ideas we use a well-known model and introduce two variants to that model. The canonical model is MacArthur’s resource consumer model (MCRM) [15], that will be referred below as the “ground model”. The variants introduce small changes to its dynamical evolution.

The MCRM describes the dynamics of *S* species abundances *N*_*i*_(*i* = 1, ‥, *S*) competing over *M* types of resources *R*_*β*_(*β* = 1, ‥, *M*). The MCRM system evolves according the following set of coupled differential equations

$$\begin{array}{c} \left\{ \begin{array}{c} \frac{dN_{i}}{dt}=N_{i}[\sum_{\beta }c_{i\beta }R_{\beta }-m_{i}]+\eta_{i}\\ \frac{dR_{\beta }}{dt}=R_{\beta }[K_{\beta }-R_{\beta }-\sum_{j}c_{j\beta }N_{j}] \end{array},\right. \end{array}$$ (1)

where *c*_*iβ*_ describes the consumption preference of species *i* for resource *β*. *m*_*i*_ is a minimum maintenance cost that must be met by species *i* for it to grow. *K*_*β*_ is the carrying capacity of resource *β*. The first equation includes a migration term *η*_*i*_ from a species pool. It will taken to be small, allowing species to invade if they have positive growth rates.

Following [15], we consider the case where resources dynamics equilibrate quickly compared to the species dynamics, so that *dR*_*β*_/*dt* ≈ 0, and therefore *R*_*β*_ = *K*_*β*_ − ∑_*j*_ *c*_*jβ*_ *N*_*j*_. Substituting this into the first equation yields

$$\begin{array}{c} \frac{dN_{i}}{dt}=N_{i}[\sum_{\beta }c_{i\beta }K_{\beta }-m_{i}-\sum_{j}\alpha_{ij}N_{j}]+\eta_{i}\;, \end{array}$$ (2)

where $\alpha_{ij}=\alpha_{ij}^{\left(r\right)}=\sum_{\beta }c_{i\beta }c_{j\beta }$, and the superscript (*r*) denotes resource-mediated interactions. This equation is now in the form of generalized Lotka-Voltera equations.

In going from Eqs (1) to (2) there is a risk, that the dynamics will pass through negative values of *R*_*β*_, which are biologically unrealistic. As shown below, all the phenomenology described using Eq (2) is also present in a model starting from Eq (1). See discussion in Sec. Theory for the onset of non-equilibrium dynamics, and S1 Text (Appendix I).

### Demonstrating the sensitivity

A key result by MacArthur [15] is that the model in Eq (2) exhibits globally stable dynamics, reaching a single fixed point independently of the system’s initial conditions. In this section we show that by a small addition of other interactions on top of the resource competition interactions described above, the system is no longer guaranteed to approach a fixed point. Instead, for a broad region of control parameters, the species’ abundances fluctuate indefinitely, as shown in Fig 1(C).

To demonstrate this phenomena we introduce the first variant of the MCRM, which includes additional “direct” species interactions, $\alpha_{ij}^{\left(d\right)}$, so that the total interaction coefficients read $\alpha_{ij}=\alpha_{ij}^{\left(r\right)}+\omega \cdot \alpha_{ij}^{\left(d\right)}$, with *ω* controlling the strength of the perturbation. These direct interactions may come as a result of many mechanisms that lie beyond the unperturbed MCRM. The important point will be to find when such additional interactions have a significant effect on the dynamics, even when they are small.

To quantify the size of the perturbation, we use the ratio of the Frobenius norms (sum of squared interaction coefficients) of the interaction matrices, setting ‖*ω* ⋅ *α*^(*d*)^‖_*F*_/‖*α*^(*r*)^‖_*F*_ = 0.05 throughout. For any given model parameters, *ω* is chosen to satisfy this condition, allowing for a comparison between results with different model parameters.

The quantities *c*_*iβ*_, *K*_*β*_, *m*_*i*_, $\alpha_{ij}^{\left(d\right)}$ that define the interactions are drawn at random, representing a generic diverse community, without any additional structure beyond that already incorporated into the resource-competition model. The parameters *c*_*iβ*_, *m*_*i*_, *K*_*β*_ are drawn independently for each value, and $\alpha_{ij}^{\left(d\right)}$ are drawn independently, except possibly a correlation between $\alpha_{ij}^{\left(d\right)}$ and $\alpha_{ji}^{\left(d\right)}$ controlling the symmetry of the direct interactions. All quantities are drawn from Gaussian distributions, parameterized by their first two moments. The results depend only on combinations of these first two moments with *S*, for example, $\sigma_{c}=\sqrt{S}\;\mathrm{std}(c_{i\beta })$ and *μ*_*c*_ = *S* mean(*c*_*iβ*_); for sparse networks *S* is replaced by the number of non-zero links per species. The definitions of all these control parameters are given in S1 Text (Appendix A).

The analytical treatment requires that *S* is large. Very strong competitive interactions, which would lead to mutual exclusion between pairs of species, are assumed to be rare in the community. A significant number of mutually exclusive pairs can lead to a different phenomenology [24].

As seen in Fig 1(C), the variant with even these small additional interactions shows persistent abundance fluctuations, even while the ground model reaches equilibrium as it always does.

In this section we provided a proof-of-principle demonstration of the sensitivity of the equilibrium to variations in the model dynamics. We now turn to show that this is in fact a robust and general phenomenon, found in a broad regime of parameters, and explain where it is expected and why.

### Theory for the onset of non-equilibrium dynamics

To understand more generally whether and when the variant of the model will reach a fixed point or a non-equilibrium state, we assume a fixed point is reached and ask whether it is stable. The basic idea is that systems are sensitive to perturbations, if the fixed point in the ground model is close to marginal stability.

Before turning to the present model, we review the results for the random Lotka-Volterra models, which in the terminology of Sec. Demonstrating the sensitivity have only “direct” interactions, $\alpha_{ij}=\alpha_{ij}^{\left(d\right)}$. Their dynamics have been studied recently [8, 9, 12, 14] (see also related results in other models [6]). A number of sharply delineated regions in parameter space, or “phases”, have been found. In one phase the system reaches a unique equilibrium. The boundary of this phase is marked by loss of stability of these fixed points. Beyond this boundary (with a sharp transition at large *S*) lies another phase, where the dynamics fail to reach a fixed point and abundances fluctuate indefinitely [14]. In a special case where the interactions are symmetric, namely *α*_*ij*_ = *α*_*ji*_, this phase is instead characterized by many possible alternative equilibria, all of which are close to marginal stability [12].

**Fig 2:**
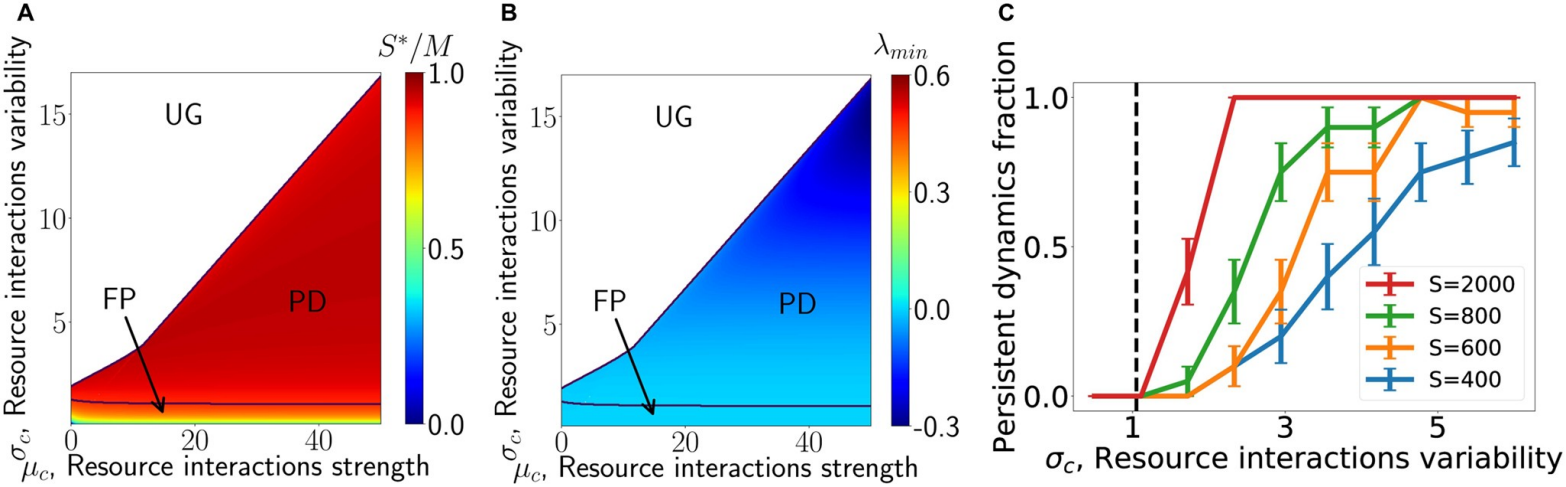
The model exhibits three phases, i.e. regions with qualitatively distinct behavior. In one, the system converges to a stable fixed point (FP), in another fixed points of the system are unstable yielding persistent dynamics (PD). In the third phase, unbounded growth (UG), species abundances grow without bound. *ω* is adjusted in order to maintain constant perturbation strength of 0.05. (A) Color map of the ratio S*/*M*, indicating how close the system is to competitive exclusion S*/*M* = 1, assuming an equilibrium reached. In the PD phase the calculation of S*/*M* is no longer valid and S*/*M* > 1 may be reached (see later sections). (B) The minimal real part of eigenvalues of the interaction matrix between coexisting species, λ_*min*_. Fixed point stability is lost at λ_*min*_ = 0, where the solid line separates the FP and PD phases. The increase in S*/*M* reduces the stability of the equilibria, triggering a transition to persistent dynamics. (C) Probability of reaching persistent dynamics along a vertical cross section of the diagrams in panels A and B, in simulations with different pool richness *S*. The transition between equilibrium and non-equilibrium outcomes becomes sharper as system size increases, and matches the theoretically predicted transition point between the phases (dashed line).

The behavior of the model variants defined here (above and in Sec. Non-linear resource intake below), bears many similarities to that of the random Lotka-Volterra models, see Fig 2. There is a unique-equilibrium phase, which is delineated by a boundary at which the equilibrium loses its stability. Beyond it, we find in simulations that the dynamics never reach a fixed point, see Fig 2(C). The case of symmetric $\alpha_{ij}^{\left(d\right)}$ is special and appears to follow the scenario in [12], see S1 Text (Appendix H). An important difference from random Lotka-Volterra models is the mechanism by which fixed points lose their stability, which we now discuss.

We describe a method of calculating the species richness and stability of the fixed points for the model variant described in Sec. Demonstrating the sensitivity. This method is exact when the system admits a unique fixed point; the loss of its stability marks the boundary of the phase. To highlight the relation between marginal stability and sensitivity to perturbations, we study the spectrum of the interaction matrix, and how it changes for the model variant described above. A different approach, using Dynamical Mean Field Theory, is possible and ultimately equivalent, and has been employed on a related model in [7].

Consider fixed points of the dynamics, i.e. abundance vectors $\vec{N}$ for which $d\vec{N}/dt=0$ in Eq (2). We are interested in the linear stability of these fixed points under perturbations. Specifically, we consider a perturbation which modifies the growth rates of the surviving species (species for which *N*_*i*_ → *c* > 0, even as the migration *η*_*i*_ → 0^+^). Eq (2) changes to *dN*_*i*_/*dt* = *N*_*i*_[… + *ξ*_*i*_] + *η*_*i*_, where $\vec{\xi }$ is a perturbation, constant in time (press perturbation). The change in the fixed point abundances of the surviving species, due to a small perturbation and at small migration *η*_*i*_ → 0^+^, is given by

$${\frac{\partial N_{i}}{\partial \xi_{j}}|}_{\vec{\xi }=0}={\left(\alpha^{*}\right)}_{ij}^{-1}.$$

Here α* is the reduced interaction matrix, comprised only from interactions between surviving species. This expression diverges when α* becomes singular, i.e. when the minimum of the real part of the eigenvalues, λ_*min*_ ≡ min{Re[Λ(α*)]}, touches zero. It signals the loss of stability of the fixed point. A fixed point is called marginally stable if λ_*min*_ → 0 when *S* → ∞.

While the MCRM only has stable or marginally stable fixed points, i.e. with λ_*min*_ ≥ 0, in the model variant λ_*min*_ can cross zero and become negative. In related models [6, 7, 14] this signals a transition to a phase with persistent dynamics. We show in simulations that this also happens here, see below (Fig 2(C)). This result can also be derived more formally, as has been done for other models [6, 7, 14].

**Fig 3:**
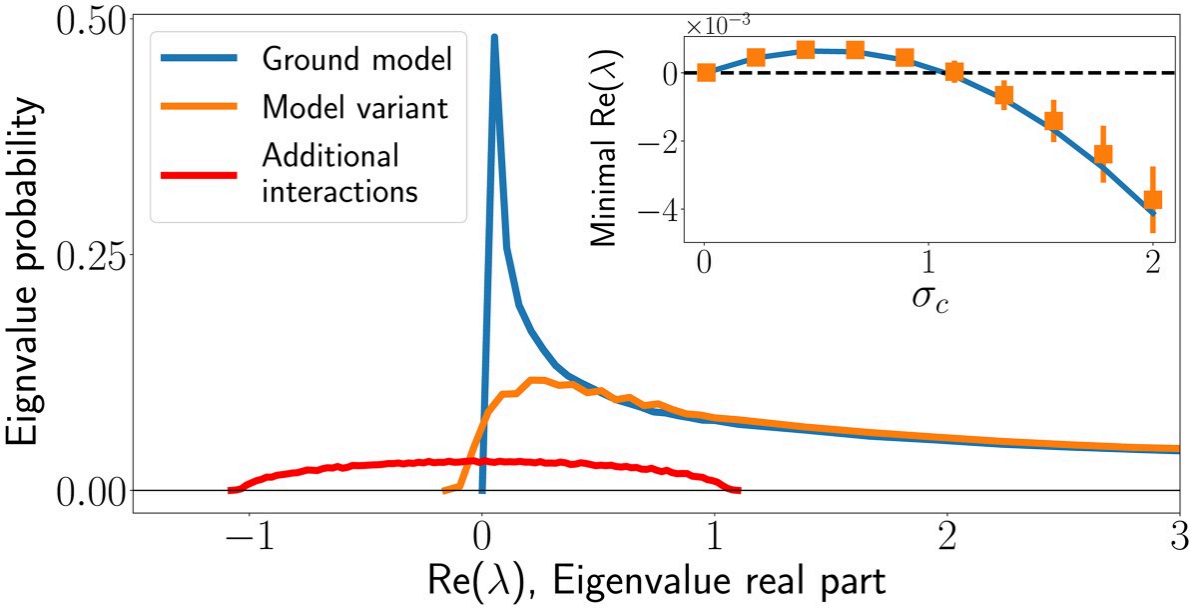
Spectrum of α*, the interaction matrix of persistent species, in the ground model (blue), and the variant with additional direct interactions (orange). The perturbation spectrum is shown in red, to illustrate its size we normalize the area under the perturbation spectrum to the size of the relative perturbation strength (0.05). (Inset) Minimal eigenvalue real part of the reduced interaction matrix α*, when varying *σ*_*c*_ at fixed *μ*_*c*_. Solid line is theoretical curve. A phase transition occurs when the minimal real part of the eigenvalues crosses λ_*min*_ = 0, from λ_*min*_ > 0 at which fixed points of the dynamical system are stable, to λ_*min*_ < 0 where all fixed points are unstable and persistent dynamics ensue. For example, here the transition happens at *σ*_*c*_ close to 1.

Close to marginality, i.e. when λ_*min*_ is zero or close to zero, even a small perturbation is able to push parts of the spectrum to λ_*min*_ < 0. This is the case for a broad region in parameter space, as we now show. In Fig 3, we show the spectrum of such an α* matrix close to a marginal fixed point. As expected, the marginal case is characterized by non-vanishing density of eigenvalues arbitrarily close to zero. When applying a small random perturbation to the marginal interaction matrix α*, for example $\alpha_{ij}^{\left(d\right)}$ described in Sec. Demonstrating the sensitivity, the spectrum is broadened and may cross zero to give eigenvalues with negative real parts, resulting in a dynamically unstable fixed point. The properties of the fixed point depend crucially on the species richness (the number of species that survive), which is a result of a balance between competition that pushes species out of the system, and migration which allow them to try and invade.

The method for calculating the spectrum consists of the following main steps: first, we find the number S* of coexisting species using the cavity method. This follows similar calculations in previous works [9, 13], and is detailed in S1 Text (Appendix C). We then calculate λ_*min*_, the minimal real part of the eigenvalues of α*, for a reduced interaction matrix with S* species. This is done using random matrix theory and detailed in S1 Text (Appendix D).

Following this method, we can predict the dynamical behavior as a function of the model parameters. We find three phases, shown in Fig 2. In the first, the system converges to a unique fixed point, independent of the initial conditions, as in Fig 1(B). In the second, the system fails to reach a fixed point, with abundances fluctuating indefinitely, as in Fig 1(C). In the third phase the abundances diverge, indicating that the model is no longer adequate in this parameter regime.

Notably, when S*/*M* ≈ 1 the unperturbed system is close to competitive exclusion and correspondingly close to marginality, and therefore the model variant with the direct interactions becomes unstable, i.e. λ_*min*_ < 0. As expected theoretically, the transition between the two behaviors is sharp when *S*, *M* are large, and happens at the theoretically predicted value of the parameters, see Fig 2(C). In less diverse systems, the transition is more gradual. Thus, our analytical method allows us to precisely predict the location of the transition between equilibrium and non-equilibrium phases, which is borne out by simulations.

The loss of stability of putative fixed points results in persistent dynamics where species invade but are then pushed back out by the instability of fixed points. This is clear in Fig 1(C).

As mentioned before, when going from Eqs (1) to (2), we have replaced the resource values *R*_*β*_ with their equilibrium values without making sure that resource values remain positive, *R*_*β*_ > 0, as expected for resource abundance. We show in S1 Text (Appendix I) that all the phenomenology described hitherto is also found in a model that starts directly from Eq (1), where resources are modeled explicitly, and prevented from going negative by migration. The main difference is that now equilibria loose their stability when S*/M* ≈ 1, where M* is the number of resources present in the community (that have not gone locally extinct). There we also show that the phase where abundances diverge can be removed by stronger limitation on growth rate at high abundances, with little effect on the transition between equilibrium and persistent dynamics.

### Non-linear resource intake

So far, we have discussed the ground model with a small addition of other interactions. This allows us to identify regions in parameter space where the ground model is sensitive to perturbations. By adding interactions that are not mediated by resource competition, the model variant can no longer be strictly interpreted as a resource-competition model. Here we consider a second variant of the ground model, which belongs to the resource-competition class, but with non-linear resource intake. There are many situations in which growth rates depend on resource availability in ways that lie outside the linear, additive MCRM model, such as in competition over essential resources [19, 25]. Here the aim is not to study the consequences of a specific non-linear mechanism, but rather to demonstrate the sensitivity of the model to such variations in the dynamical rules.

We find that much like the model variant discussed in previous sections, here too the dynamics are sensitive to changes to the ground model, with fixed points turning into persistent abundance fluctuations, in much the same parameter regions as found previously.

The second variant to the ground model, Eq (2), is different from the ground model in the way that different resources translate into the growth rate of the consumer. Whereas in Eq (2) the growth rate is a linear combination of the resource values *R*_*β*_, here we use a non-linear function. We choose a non-linear consumption function $h(R)=\frac{1}{w}\tanh(wR)$ with control parameter *w*. With this consumption function, the dynamical equations read:

$$\begin{array}{c} \left\{ \begin{array}{cc} \frac{dN_{i}}{dt} & =N_{i}[\sum_{\beta }\frac{1}{w}\tanh(wc_{i\beta }R_{\beta })-m_{i}]+\eta_{i}\\ R_{\beta } & =K_{\beta }-\sum_{j}c_{j\beta }N_{j} \end{array}\right. \end{array}$$ (3)

**Fig 4:**
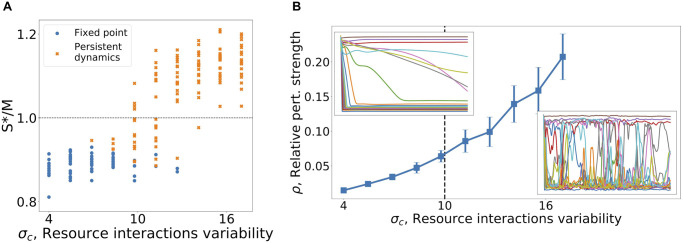
The transition between the equilibrium and persistent dynamics phases in a model with non-linear resource intake. (A) As model parameters are changed (here varying *σ*_*c*_) the fraction of systems exhibiting persistent dynamics varies. Communities reaching a fixed-point must satisfy S*/*M* < 1 due to competitive exclusion, but in the persistent dynamics regime can sustain more coexisting species, with S*/*M* > 1. (B) The relative perturbation strength, *ρ*, quantifies how much the growth-rate as a function of resource availability deviates from the weighted linear sum (see text for definition). *ρ* changes as model parameters are changed, and is measured in simulations. Persistent non-equilibrium dynamics already found for *ρ* ≳ 0.06 marked by dashed vertical line.

The parameter *w* allows us to tune the deviation from the ground model. For small values of *w*, *h*(*R*) ≃ *R* so the non-linear effects become small and the equations reduce to the ground model, Eq (2). For finite *w* non-linear effects may be significant. We quantify the deviation from linearity explored by the dynamics by the relative perturbation strength $\rho =1-{\langle \frac{\text{tanh}(wc_{i\beta }{\bar{R}}_{B})}{wc_{i\beta }{\bar{R}}_{B}}\rangle }_{i\beta }$, where 〈‥〉_*iβ*_ is the average over species and resources, and $\bar{R_{\beta }}$ denotes the time average of the resource abundance *R*_*β*_ (taken over a window of Δ*t* = 1000). We find that even a rather small value of *ρ* is sufficient to induce a transition to dynamical persistence, see Fig 4, where the transition occurs at *ρ* ∼ 0.06. Again, this demonstrates how the system’s dynamics may be sensitive to small changes in the equations governing the model, in this case in a variant that is itself strictly a resource competition model.

### Species abundance distribution and diversity

Above, we saw how communities near marginal stability are sensitive to small variations in the dynamical rules, either due to additional interactions, or changes to the functional form of the interactions. Here we show that these changes can allow the diversity to go well above the number of resources. This is made possible by the persistent dynamics, which are no longer bound by the competitive exclusion principle.

The competitive exclusion principle [26] states that for models describing an ecological community of *S* species relying on *M* limiting resources, no stable fixed points with *M* < S* exist. Briefly, the core of the argument is that any fixed point with *M* < S* would imply a degenerate Jacobian matrix with rank *M* or less. This kind of fixed point can be marginally stable, but not stable. The second variant of the model, Eq (3), satisfies the conditions for this principle to hold, so the diversity of stable equilibria is bound by *M*.

**Fig 5:**
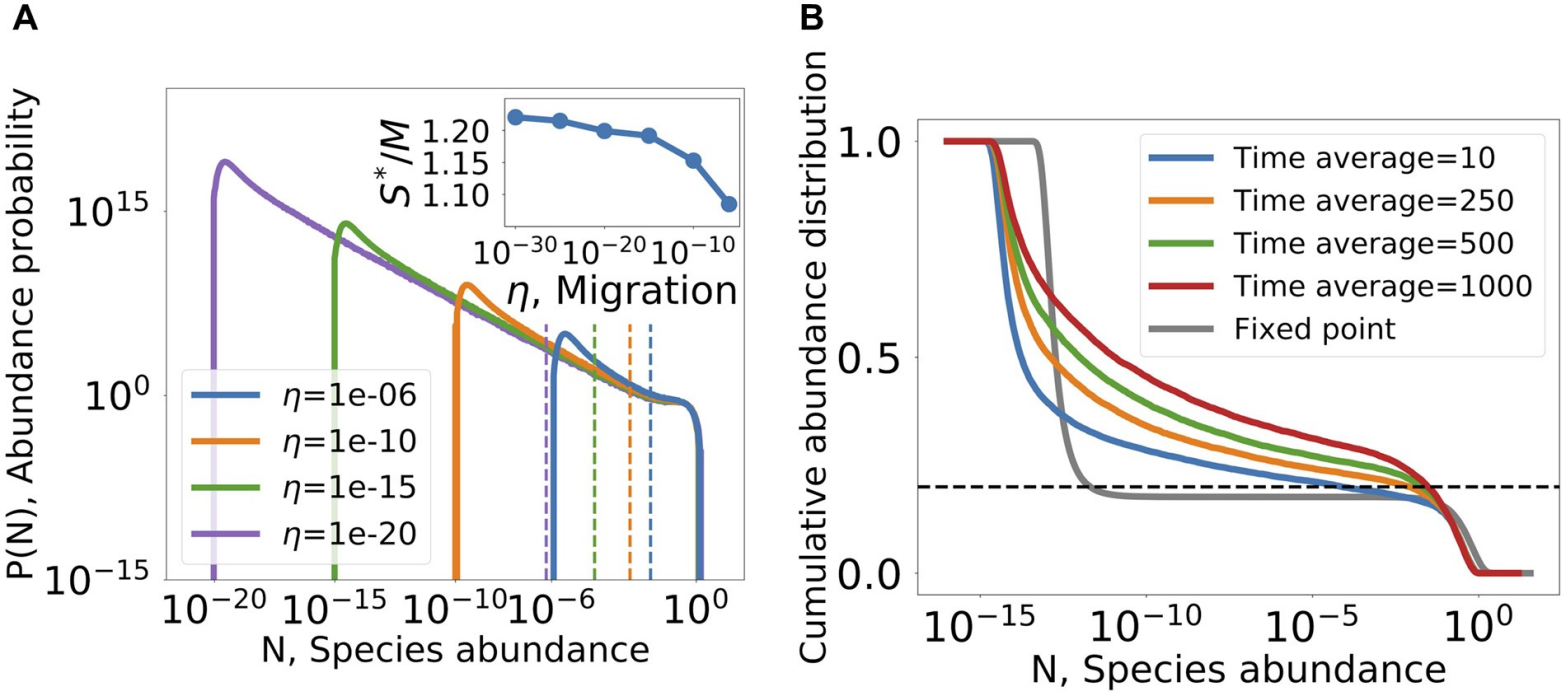
Diversity above competitive exclusion in the non-linear resource competition model, see Sec. Non-linear resource intake. (A) Abundance distribution for different values of migration. The area to the right of the vertical lines hold exactly *M* species. The rest of the distribution, below the line, accounts for species above competitive exclusion. Inset: the total number of coexisting species normalized by number of resources, with values above one indicate crossing of competitive exclusion. (B) Due to the abundance fluctuations, averaging abundances over a time window pushes the distribution of abundance upwards due to fluctuations. The cumulative abundance distribution is shown, defined as $F(N)=\frac{1}{S}\sum_{i}1_{\left[{\bar{N}}_{i}>N\right]}.$ The dashed line is the competitive exclusion bound, *M*/*S*. For comparison, the distribution at a fixed point is given, showing that the number of species at high abundance(*N* ≳ 10^−3^ does not reach this bound, and the rest of the species are at low abundances, only supported by migration.

As an example we look at the second model variant, as defined in Sec. Non-linear resource intake. Long-time simulations of the persistent dynamics show that the species abundance distribution converges to a stationary form that can be decomposed into a power law at intermediate abundances, and other functions at the highest and lowest abundances, see Fig 5(A):

$$\begin{array}{c} P\left(N\right)=\{\begin{array}{cc} P_{high}(N) & N_{u}<N\\ cN^{-(\nu +1)} & \eta ≲N<N_{u}\;.\\ P_{low}(N) & N≲\eta \end{array} \end{array}$$ (4)

Here *N*_*u*_ is a constant, and *c* is set by the normalization *∫* *P*(*N*)*dN* = 1. From the simulations, *ν* is not far from zero when *η* → 0 (*ν* ≈ 0.02 in Fig 5, and similar for other parameter sets, see S1 Text (Appendix B)).

We first ask about the instantaneous species richness, namely the fraction of species that are not at the migration floor (say, above 100*η*). By integrating *P*(*N*) in Eq (4) one finds that the fraction of the species above the migration floor approaches a finite number when *η* → 0^+^, for details see S1 Text (Appendix E). This number can be larger than *M*, and in fact is so in the example shown in Fig 5, reaching about 20% above *M* (see inset in Fig 5(A)). This is also found in results of many simulations at different parameters, see Fig 4(A). In other words, a finite fraction of the species coexist above the competitive exclusion limit even when migration is very small. This is possible since the community is not in a fixed point, and so is not bound by the competitive exclusion principle.

If the species abundance is measured by integrating over a finite-time window, see Fig 5, the abundances shift to higher values as the time window grows, indicating that species have periods of time with high abundance. This leads to a growth in the abundance *N*_*CE*_ above which there are exactly *M* species with higher abundances.

In [23], chaotic dynamics where studied in Lotka-Volterra equations with random interactions coefficients, and the existence of of time periods with high abundance have been reported, as well as a power law like in Eq (4) (albeit with a different exponent). The relation of these results to the present resource-competition model are an interesting question for future research.

## Discussion and conclusions

In this work we have shown how diverse ecological communities with resource-competition interactions may display non-equilibrium dynamics. This turns out to be closely related to the ratio of realized species diversity to the number of resources, S*/*M*. When this number is larger than one, fixed-points are either unstable or marginally stable, as expected by the competitive exclusion principle. If they are unstable, the system is pushed away from fixed points, and abundances forever fluctuate. While marginal-stable fixed points are in principle possible, they are structurally unstable under variations in the model, such as non-linearities that destabilize the fixed points.

### Comparison with random Lotka-Volterra models

This picture bridges a gap to the behavior of high-dimensional models where interactions are sampled at random without a specified mechanism. In the notation of Sec. Demonstrating the sensitivity, this corresponds to having $\alpha_{ij}=\alpha_{ij}^{\left(d\right)}$ only. These models show a phase with persistent dynamics [6–9, 12, 14], in contrast to resource-competition which have thus far only shown relaxation to equilibrium in highly diverse communities [10, 13]. We find that the generic phase-diagram is in fact much more similar, with a transition to non-equilibrium dynamics when the variability in interaction strengths is high enough (compare, for example, Fig 2 with the phase diagram in [9]).

One difference is that here, the symmetry of the interactions can be very high and still lead to non-equilibrium dynamics. For example, in the model with added direct interactions (Sec. Demonstrating the sensitivity), the total interactions are very close to symmetric, with corr(α_*ij*_, α_*ji*_) = 0.997. In random Lotka-Volterra models, dynamics at a comparable level of symmetry would typically relax to equilibria. This highlights the importance of certain structures in the interaction network on dynamics.

### Predictions

How can the behavior presented in this work be identified in natural or experimental communities? The dynamical outcome will depend on the following considerations:

- Is the community isolated; under migration from a regional species pool; or part of a meta-community?
- The ratio of realized species diversity to the number of resources (S*/*M*).
- Is the realized diversity S* high enough for high-diversity effects to show?

Consider first a single well-mixed system with continuous migration from a species pool, which was the focus of previous sections. In such a setting, dynamics either a relax to single uninvadable equilibria or reach persistent fluctuations. Which of these two possibilities is realized depends on the system parameters: the realized species diversity (S*) is set by the balance between extinctions due to competition and species able to invade. If fixed points at this diversity are unstable, persistent fluctuations will result—this is the Persistent Dynamics phase in Fig 2. As shown in Fig 2, it is attained when there is sufficient variability in the interactions, mediated for example by a broad distribution of consumption preferences (high *σ*_*c*_).

We turn to a single well-mixed community that is isolated (no migration, *η*_*i*_ = 0). Here species may go extinct due to large abundance fluctuations, without being able to invade again. Extinctions may then lead to equilibria even when non-equilibrium dynamics are expected with migration, see S1 Text (Appendix G). The difference is that these equilibria can be invaded by species from the species pool. Importantly, in these conditions *all* fixed points are invadable, as uninvadable ones would translate to equilibria in the presence of migration. If there are now isolated migration events from the species pool that are well-separated in time (for example, at low migration rates, or in experiments where species are re-introduced) the equilibria will be punctuated by migration events that change the community composition [27].

An explicit spatial dimension, such as a meta-community in which several well-mixed systems are coupled by migration, again changes the phenomenology. A meta-community can exhibit persistent fluctuations, even if it is isolated from any outside species pool, thus allowing species to go extinct within it. Still, the remaining species might continue to fluctuate for extremely long times without inducing extinctions. This has been shown recently for many-species meta-communities with random Lotka-Volterra interactions in [22, 23]. An example simulation, provided as a proof-of-principle, is given in S1 Text (Appendix G). The conditions for non-equilibrium dynamics to persist depend on additional parameters including the migration rates and the number of communities in the meta-community. A fuller account of this effect is an interesting direction for future research.

Finally, we note that the non-equilibrium dynamics discussed in this work apply to communities with many species and resources or niches. Simulations indicate that dynamical fluctuations appear when there are tens of species in the community or more; communities with fewer species may instead relax to equilibria.

We have looked at interactions without any structure beyond resource competition, such as different functional groups or higher trophic levels. More complex interaction networks may possibly be approached by combining “fully random” interactions used here, in a modular fashion [11]; the implications to dynamics are an interesting subject for future research.

High-dimensional ecological dynamics are, in some respects, qualitatively different from their low-dimensional counterparts. Here we classified possible scenarios for the dynamics of resource-competition communities, and provided predictions for each scenario. We hope it may help in guiding future theoretical works, observations and experiments on high-diversity communities.

## Supporting information

S1 Text Appendices: Details of models, simulations and theory. (PDF)

## References

[1] Armstrong Robert A. and McGehee Richard. Competitive exclusion. *The American Naturalist*, 115(2):151–170, 1980 10.1086/283553

[2] Huisman Jef and Weissing Franz J. Biodiversity of plankton by species oscillations and chaos. *Nature*, 402(6760):407–410, 1999 10.1038/46540

[3] Turchin Peter and Taylor Andrew D. Complex dynamics in ecological time series. *Ecology*, 73(1):289–305, 1992 10.2307/1938740

[4] Benincà Elisa, Huisman Jef, Heerkloss Reinhard, Jöhnk Klaus D., Branco Pedro, Van Nes Egbert H., Scheffer Marten, and Ellner Stephen P. Chaos in a long-term experiment with a plankton community. *Nature*, 451(7180):822–825, 2 2008 10.1038/nature06512 18273017

[5] Schippers Peter, Verschoor Antonie M., Vos Matthijs, and Mooij Wolf M. Does “supersaturated coexistence” resolve the “paradox of the plankton”? *Ecology Letters*, 4(5):404–407, 2001 10.1046/j.1461-0248.2001.00239.x

[6] Opper Manfred and Diederich Sigurd. Phase transition and 1/f noise in a game dynamical model. *Physical review letters*, 69(10):1616, 1992 10.1103/PhysRevLett.69.1616 10046267

[7] Yoshino Yoshimi, Galla Tobias, and Tokita Kei. Statistical mechanics and stability of a model eco-system. *Journal of Statistical Mechanics: Theory and Experiment*, 2007(09):P09003, 2007 10.1088/1742-5468/2007/09/P09003

[8] Kessler David A. and Shnerb Nadav M. Generalized model of island biodiversity. *Physical Review E*, 91(4):042705, 2015 10.1103/PhysRevE.91.042705 25974525

[9] Bunin Guy. Ecological communities with Lotka-Volterra dynamics. *Physical Review E*, 95(4), 4 2017 10.1103/PhysRevE.95.042414 28505745

[10] Tikhonov Mikhail and Monasson Remi. Collective Phase in Resource Competition in a Highly Diverse Ecosystem. *Physical Review Letters*, 118(4), 1 2017 10.1103/PhysRevLett.118.048103 28186794

[11] Barbier Matthieu, Arnoldi Jean-François, Bunin Guy, and Loreau Michel. Generic assembly patterns in complex ecological communities. *Proceedings of the National Academy of Sciences*, 115(9):2156–2161, 2 2018 10.1073/pnas.1710352115 29440487 PMC5834670

[12] Biroli Giulio, Bunin Guy, and Cammarota Chiara. Marginally stable equilibria in critical ecosystems. *New Journal of Physics*, 20(8):083051, 8 2018 10.1088/1367-2630/aada58

[13] Advani Madhu, Bunin Guy, and Mehta Pankaj. Statistical physics of community ecology: A cavity solution to MacArthur’s consumer resource model. *Journal of Statistical Mechanics: Theory and Experiment*, 2018(3):033406, 3 2018 10.1088/1742-5468/aab04e 30636966 PMC6329381

[14] Roy F, Biroli G, Bunin G, and Cammarota C. Numerical implementation of dynamical mean field theory for disordered systems: Application to the Lotka–Volterra model of ecosystems. *Journal of Physics A: Mathematical and Theoretical*, 52(48):484001, 11 2019 10.1088/1751-8121/ab1f32

[15] Arthur Robert Mac. Species packing, and what competition minimizes. *Proceedings of the National Academy of Sciences*, 64(4):1369–1371, 1969 10.1073/pnas.64.4.1369 PMC223294 16591810

[16] Arthur Robert Mac. Species packing and competitive equilibrium for many species. *Theoretical Population Biology*, 1(1):1–11, 5 1970 10.1016/0040-5809(70)90039-0 5527624

[17] Wenping Cui, Robert Marsland III, and Pankaj Mehta. The effect of resource dynamics on species packing in diverse ecosystems. arXiv:1911.02595 [cond-mat, physics:physics, q-bio], November 2019. 10.1103/PhysRevLett.125.048101 PMC8999492 32794828

[18] Landmann Stefan and Engel Andreas. Systems of random linear equations and the phase transition in MacArthur⃥textquotesingles resource-competition model. *EPL (Europhysics Letters)*, 124(1):18004, 11 2018 10.1209/0295-5075/124/18004

[19] León Jesús Alberto and Tumpson Daniel B. Competition between two species for two complementary or substitutable resources. *Journal of Theoretical Biology*, 50(1):185–201, 3 1975 10.1016/0022-5193(75)90032-6 1127957

[20] Posfai Anna, Taillefumier Thibaud, and Wingreen Ned S. Metabolic Trade-Offs Promote Diversity in a Model Ecosystem. *Physical Review Letters*, 118(2):028103, 1 2017 10.1103/PhysRevLett.118.028103 28128613 PMC5743855

[21] Roy Shovonlal and Chattopadhyay J. Towards a resolution of ‘the paradox of the plankton’: A brief overview of the proposed mechanisms. *Ecological Complexity*, 4(1):26–33, 3 2007 10.1016/j.ecocom.2007.02.016

[22] Roy Felix, Barbier Matthieu, Biroli Giulio, and Bunin Guy. Can endogenous fluctuations persist in high-diversity ecosystems? *bioRxiv*, page 730820, 8 2019.

[23] Pearce Michael T., Agarwala Atish, and Fisher Daniel S. Stabilization of extensive fine-scale diversity by spatio-temporal chaos. *bioRxiv*, page 736215, 2019. 10.1073/pnas.1915313117 PMC7322069 32518107

[24] Guy Bunin. Directionality and community-level selection. Preprint, bioRxiv:484576, December 2018.

[25] Huisman Jef and Weissing Franz. Oscillations and chaos generated by competition for interactively essential resources: Competitive chaos. *Ecological Research*, 17(2):175–181, 3 2002 10.1046/j.1440-1703.2002.00477.x

[26] McGehee Richard and Armstrong Robert A. Some mathematical problems concerning the ecological principle of competitive exclusion. *Journal of Differential Equations*, 23(1):30–52, 1977 10.1016/0022-0396(77)90135-8

[27] Law Richard and Morton R. Daniel. Alternative permanent states of ecological communities. *Ecology*, 74(5):1347–1361, 1993 10.2307/1940065

