# Supplementary material for: Dynamical persistence in high-diversity resource-consumer communities
Source: PLoS Comput Biol. 2020 Oct 12;16(10):e1008189. doi: 10.1371/journal.pcbi.1008189 (PMC7581001; doi:10.1371/journal.pcbi.1008189)
Supplement: S1 Text — (PDF) [file pcbi.1008189.s001.pdf]

Supporting Information for:
Dynamical persistence in high-diversity resource-consumer communities

Itay Dalmedigos, Guy Bunin

Appendix A: Basic setup

Each instance of the first model variant, defined in Sec. II B, requires setting the values of the quantities $\left\{c_{i\beta},\alpha_{ij}^{(d)},m_i,K_\beta\right\}$, referred to here as the disorder parameters. They are fixed from the control parameters $\{S,M,\mu_c,\sigma_c,\mu_d,\sigma_d,\gamma,\mu_m,\sigma_m,\mu_K,\sigma_K\}$ as follows.

Let us denote by $\langle X\rangle$ the expectation value of random variable X. All results cited in the paper at high-diversity depend only on the first and second moment of the system disorder parameters distribution. The means and variances of these are given by $\langle c_{i\beta}\rangle=\mu_c/S$, $\left\langle\left(c_{i\beta}-\langle c_{i\beta}\rangle\right)^2\right\rangle=\sigma_c^2/S$, $\langle m_i\rangle=m$, $\left\langle\left(m_i-\langle m_i\rangle\right)^2\right\rangle=\sigma_m^2$, $\langle K_\beta\rangle=K$, $\left\langle\left(K_\beta-\langle K_\beta\rangle\right)^2\right\rangle=\sigma_K^2$, and the direct interactions by $\left\langle\alpha_{ij}^{(d)}\right\rangle=\mu_d/S$, $\left\langle\left(\alpha_{ij}^{(d)}-\left\langle\alpha_{ij}^{(d)}\right\rangle\right)^2\right\rangle=\sigma_d^2/S$ and $\mathrm{corr}\left(\alpha_{ij}^{(d)},\alpha_{ji}^{(d)}\right)=\gamma$ with $-1\le\gamma\le1$. All other cumulants are set to zero. This definition of the parameters ensures that the abundance distribution $P(N_i)$ and the fraction of persistent species have a finite, well-defined limit as S,M are taken to be large. In other words, in that limit all results will only depend on these control parameter combinations, e.g. on $\mu_c=S\langle c_{i\beta}\rangle$ rather than on $S,\langle c_{i\beta}\rangle$ separately. The same results are obtained for a sparse interaction matrix with C non-zero links per species, as long as $1\ll C$. In that case, which includes the case $C=S$ above, the moments are rescaled by C rather than S, e.g. $\langle c_{i\beta}\rangle\doteq\mu_c/C$ instead of $\langle c_{i\beta}\rangle=\mu_c/S$.

To simplify the notation below, it is useful to separate quantities into mean and random deviation from the mean

$$c_{i\beta}\equiv\frac{\mu_c}{S}+\sigma_c d_{i\beta}\ ;\ \langle d_{i\beta}\rangle=0\ ;\ \langle d_{i\alpha}d_{j\beta}\rangle=\frac{\delta_{ij}\delta_{\alpha\beta}}{S}$$

$$\alpha_{ij}^{(d)}=\frac{\mu_d}{S}+\sigma_d a_{ij}\ ;\ \langle a_{ij}\rangle=0\ ;\ \left\langle a_{ij}^2\right\rangle=\frac{1}{S}\ ;\ \langle a_{ij}a_{ji}\rangle=\frac{\gamma}{S}$$

$$K_\beta=K+\delta K_\beta\ ;\ \langle K_\beta\rangle=K\ ;\ \langle\delta K_\alpha\delta K_\beta\rangle=\delta_{\alpha\beta}\sigma_K^2$$

$$m_i=m+\delta m_i\ ;\ \langle m_i\rangle=m\ ;\ \langle\delta m_i\delta m_j\rangle=\delta_{ij}\sigma_m^2$$

Note that the 'd' in $\alpha^{(d)}$ stands for the *direct* part of the interaction between species, and has nothing to do with the quantity $d_{i\beta}$, representing the random deviation from the mean of the entry $c_{i\beta}$.

With these definitions the first variant, Eq. (2), can be written as

$$\begin{cases}\frac{dN_i}{dt}=N_i\left[g+\sigma_c\sum_\beta d_{i\beta}R_\beta-\omega\sigma_d\sum_j a_{ij}^{(d)}N_j-\delta m_i\right]+\eta_i\\ R_\beta=K^{eff}+\delta K_\beta-\sigma_c\sum_j d_{j\beta}N_j\end{cases}$$

where

$$\langle R\rangle=\frac{1}{M}\sum_\alpha R_\alpha\quad\langle N\rangle=\frac{1}{S}\sum_j N_j$$

$$g=\mu_c\frac{M}{S}\langle R\rangle-\omega\mu_d\langle N\rangle-m$$

$$K^{eff}=K-\mu_c\langle N\rangle$$

This form of the equations will be useful below, in Appendix C.

Appendix B: Model definition, parameters and simulation details

Differential equations were integrated using a Radau integrator implemented in Python's Scipy package. Absolute integration tolerance is set to $atol = 0.1\eta$, where η is the migration strength. Initial conditions of species abundances are drawn from uniform distribution over $[0, 1]$. Perturbation strength is controlled using ω to satisfy $\left\|\omega \cdot \alpha^{(d)}\right\|_F / \left\|\alpha^{(r)}\right\|_F = 0.05$ throughout. Simulation parameters are summarized in Table I.

A Python code that run simulations of the ground model and its two variants, with example parameters for the Fixed Point and Persistent Dynamics phases, is given in: https://github.com/Itaydal/crm-chaos.

	S	M	S/M	μ_c	σ_c	μ_d	σ_d	K	m	η	w (Sec. II D)
Fig. 1 (B)	800	160	5	20	0.5	10	20	2	0.2	10^{-15}	-
Fig. 1 (C)	800	160	5	20	4	10	20	2	0.2	10^{-15}	-
Fig. 2	1000	200	5	30	10	10	20	2	0.2	-	-
Fig. 3 (A,B)	∞	∞	5	Varying	Varying	10	20	2	0.2	-	-
Fig. 3 (C)	Varying	Varying	5	20	Varying	10	20	2	0.2	10^{-15}	-
Fig. 4	800	160	5	30	Varying	-	-	2	0.2	10^{-13}	$0.05 \cdot S$
Fig. 5	800	160	5	50	20	-	-	5	0.2	Varying	$0.05 \cdot S$
Fig. A	800	160	5	20	4	10	20	2	0.2	10^{-15}	-
Fig. B	800	56	14.3	20	4	10	20	2	0.2	10^{-15}	-
Fig. D	800	160	5	20	4	10	20	2	0.2	0	-
Fig. E	Varying	Varying	2	40	23	10	10	3	5	10^{-15}	-
Fig. F	∞	∞	2	40	Varying	10	10	3	5	10^{-15}	-
Fig. G	800	120	6.7	50	Varying	10	2	2	0.2	10^{-15}	-
Fig. H	400/800	60/120	6.7	50	Varying	10	2	2	0.2	10^{-15}	-

Table I: Simulation parameters used to create each of the figures. The parameters $\gamma, \sigma_K, \sigma_m$ are set to zero throughout.

Appendix C: Cavity equations

To study the properties of a typical fixed point of the model, we use a variant of the cavity method [1–6]. It proceeds by adding one new species and one new resource, along with newly sampled interactions between it and the rest of the system, creating an $S+1$ species system with $M+1$ resources. Then, by comparing the properties of a typical species of the old system with those of the newly added species and resource we get self-consistent equations for the macroscopic variables $\phi, \langle N\rangle, \langle N^2\rangle$ where $\phi = S^*/S$ is the fraction of living species, together with the properties of the resources.

Solving these self-consistent Eq. (C1) for range of parameters allows us to derive the phase diagram in Fig. 3. In particular, the distinction between stable and non-equilibrium phases is done by solving for ϕ for some choice of control parameters, this determines the distribution of reduced interaction matrices, in Appendix D we calculate it's stability. The transition into the unbounded growth phase is found at the divergence of $\langle N\rangle$.

1. Deriving species and resource distributions using cavity method

Introducing to the system new resource and species R_0 and N_0

$$\begin{aligned}\frac{1}{N_i}\frac{dN_i}{dt} =& \Big[g + \sigma_c \sum_\alpha d_{i\alpha} R_\alpha + \sigma_c d_{i0} R_0 \\ & -\delta m_i - \omega\sigma_d \sum_j a_{ij} N_j - \omega\sigma_d a_{i0} N_0\Big] \\ R_\alpha =& K^{eff} - \sigma_c \sum_j d_{j\alpha} N_j + \delta K_\alpha - \sigma_c d_{0\alpha} N_0\end{aligned}$$

and the corresponding equations for R_0 and N_0 are

$$\begin{aligned}\frac{1}{N_0}\frac{dN_0}{dt} =& \Big[g + \sigma_c \sum_\alpha d_{0\alpha} R_\alpha + \sigma_c d_{00} R_0 \\ & -\delta m_0 - \omega\sigma_d \sum_j a_{0j} N_j - \omega\sigma_d a_{00} N_0\Big] \\ R_0 =& K^{eff} - \sigma_c \sum_j d_{j0} N_j + \delta K_0 - \sigma_c d_{00} N_0\end{aligned}$$

Denote the steady-state value of a quantity X by $\bar{X}$, also denote by $\bar{X}_{\setminus 0}$ the steady-state value of X in the absence

of the resource and species $'0'$.

Then we can define the following susceptibilities

$$\chi_{i\beta}^{(N)} = \frac{\partial \bar{N}_i}{\partial K_\beta}\,; \qquad \chi_{\alpha\beta}^{(R)} = \frac{\partial \bar{R}_\alpha}{\partial K_\beta}$$
$$\nu_{ij}^{(N)} = \frac{\partial \bar{N}_i}{\partial m_j}; \qquad \nu_{\alpha j}^{(R)} = \frac{\partial \bar{R}_\alpha}{\partial m_j}$$

Since addition of single resource and species is a small (order S^{-1}) perturbation we can write

$$\bar{N}_i = \left[\bar{N}_{i\backslash 0} - \sigma_c \sum_\beta \chi_{i\beta}^{(N)} d_{0\beta} N_0 \right.$$
$$\left. - \sum_j \nu_{ij}^{(N)} \left(\sigma_c d_{j0} R_0 - \omega \sigma_d a_{j0} N_0\right)\right]$$
$$\bar{R}_\alpha = \left[\bar{R}_{\alpha\backslash 0} - \sigma_c \sum_\beta \chi_{\alpha\beta}^{(R)} d_{0\beta} N_0 \right.$$
$$\left. - \sum_j \nu_{\alpha j}^{(R)} \left(\sigma_c d_{j0} R_0 - \omega \sigma_d a_{j0} N_0\right)\right]$$

We can now plug in these expressions into the steady-state equations for N_0 and R_0. By taking leading order contributions to S^{-1}, and take expectation value over expressions we get

$$0 = \bar{N}_0 \left[g - \frac{\sigma_c^2}{S} \sum_\alpha \chi_{\alpha\alpha}^{(R)} N_0 - \omega^2 \sigma_d^2 \frac{\gamma}{S} \sum_j \nu_{jj}^{(N)} N_0 \right.$$
$$\left. + \sigma_c \sum_\alpha d_{0\alpha} \bar{R}_{\alpha\backslash 0} - \omega \sigma_d \sum_j a_{0j} \bar{N}_{j\backslash 0} - \delta m_0 \right]$$

Notice that, to leading order in S^{-1}, as sum of weakly interacting terms we can model the expression $\sigma_c \sum_\alpha d_{0\alpha} \bar{R}_{\alpha\backslash 0} - \omega \sigma_d \sum_j a_{0j} \bar{N}_{j\backslash 0} - \delta m_0$ as a Gaussian random field with mean 0 and variance

$$\sigma_g^2 = \sigma_c^2 \frac{M}{S} q_R + \omega^2 \sigma_d^2 q_N + \sigma_m^2$$

where

$$q_N = \frac{1}{S} \sum_j \bar{N}_{j\backslash 0}^2 \qquad q_R = \frac{1}{M} \sum_\alpha \bar{R}_{\alpha\backslash 0}^2$$

Let z_N be a Gaussian random field with zero mean and unit variance, and define the average susceptibilities

$$\chi = \frac{1}{M} \sum_\alpha \chi_{\alpha\alpha}^{(R)} \qquad \nu = \frac{1}{S} \sum_j \nu_{jj}^{(N)}$$

As there is no difference between species '0' and the rest, we can emit the subscript '0' to and write the equation the fixed point abundance distribution

$$0 = \bar{N} \left[g - \left(\sigma_c^2 \frac{M}{S} \chi + \omega^2 \sigma_d^2 \gamma \nu\right) \bar{N} + \sigma_g z_N\right]$$

Following similar procedure for the resources yields

$$0 = \bar{R}\left[K^{eff} - \left(1 - \sigma_c^2 \nu\right) \bar{R} + \sigma_{K^{eff}} z_R\right]$$

$$\sigma_{K^{eff}}^2 = \sigma_K^2 + \sigma_c^2 q_N$$

We can solve these equations and get

$$\bar{N} = \frac{\max\left[0, g + \sigma_g z_N\right]}{\frac{M}{S} \sigma_c^2 \chi + \omega^2 \gamma \sigma_d^2 \nu}$$

$$\bar{R} = \frac{K^{eff} + \sigma_{K^{eff}} z_R}{1 - \sigma_c^2 \nu}$$

2. Self consistent equations

At this stage, our aim is to solve for $\{\phi, \langle N\rangle, \langle R\rangle, q_N, q_R, \chi, \nu\}$ for a given set of control parameters $\{S, M, K, \sigma_K, m, \sigma_m, \mu_c, \sigma_c, \mu_d, \sigma_d, \gamma, \omega\}$. To that end, it is helpful to define

$$\Delta_g = \frac{g}{\sigma_g} = \frac{\mu_c \frac{M}{S} \langle R\rangle - \omega \mu_d \langle N\rangle - m}{\sqrt{\sigma_c^2 \frac{M}{S} q_R + \omega^2 \sigma_d^2 q_N + \sigma_m^2}}$$

and the function

$$w_j(\Delta) = \int_{-\Delta}^{\infty} \frac{dz}{\sqrt{2\pi}} e^{-\frac{z^2}{2}} (z + \Delta)^j$$

note that for $y = \max\left[0, \frac{a + c\cdot z}{b}\right]$ with z Gaussian random variable we have that

$$\langle y^j \rangle = \left(\frac{c}{b}\right)^j \int_{-\frac{a}{c}}^{\infty} \frac{dz}{\sqrt{2\pi}} e^{-\frac{z^2}{2}} \left(z + \frac{a}{c}\right)^j = \left(\frac{c}{b}\right)^j w_j\left(\frac{a}{c}\right)$$

Taking the first two moments of the distributions $\bar{N}$ and $\bar{R}$, leads to the set of set consistent equations

$$\begin{aligned}
\phi &= w_0\left(\Delta_g\right) \\
\langle N\rangle &= \left(\frac{\sigma_g}{\frac{M}{S}\sigma_c^2\chi+\omega^2\gamma\sigma_d^2\nu}\right)w_1\left(\Delta_g\right) \\
\langle R\rangle &= \frac{K^{eff}}{1-\sigma_c^2\nu}=\chi K^{eff} \\
q_N=\left\langle N^2\right\rangle &= \left(\frac{\sigma_g\nu}{\frac{M}{S}\sigma_c^2\chi+\omega^2\gamma\sigma_d^2\nu}\right)^2 w_2\left(\Delta_g\right) \\
q_R=\left\langle R^2\right\rangle &= \chi^2\left[\sigma^2_{K^{eff}}+\left(K^{eff}\right)^2\right] \\
\nu &= \left\langle\frac{\partial\bar{N}}{\partial m}\right\rangle=-\frac{\phi_S}{\frac{M}{S}\sigma_c^2\chi+\omega^2\gamma\sigma_d^2\nu} \\
\chi &= \left\langle\frac{\partial\bar{R}}{\partial K}\right\rangle=\frac{1}{1-\sigma_c^2\nu}
\end{aligned} \tag{C1}$$

The expressions for ν and χ are derived by differentiating abundances distributions N, R with respect to m and K respectively and taking their expectation values.

To avoid singularities at the diverging phase ($\langle N\rangle\to\infty$) we define $h=\frac{1}{\langle N\rangle}$, $q_n=\frac{\langle N^2\rangle}{\langle N\rangle^2}=\frac{q_N}{\langle N\rangle^2}$. With these variables the self consistent equations read

$$\begin{aligned}
\phi &= w_0\left(\Delta_g\right) \\
h &= -\frac{1}{\nu\sigma_g}\frac{w_0\left(\Delta_g\right)}{w_1\left(\Delta_g\right)} \\
\langle R\rangle &= \frac{1}{h}\chi\left(Kh-\mu_c\right) \\
q_n &= h^2\left(\frac{\sigma_g\nu}{\phi_S}\right)^2 w_2\left(\Delta_g\right)=\frac{w_2\left(\Delta_g\right)}{\left[w_1\left(\Delta_g\right)\right]^2} \\
q_R &= \frac{1}{h^2}\chi^2\left[\left(K^2+\sigma_K^2\right)h^2-2\mu_c Kh+\sigma_c^2q_n+\mu_c^2\right] \\
\nu &= -\frac{\phi_S}{\frac{M}{S}\sigma_c^2\chi+\omega^2\gamma\sigma_d^2\nu} \\
\chi &= \frac{1}{1-\sigma_c^2\nu}
\end{aligned}$$

At this stage one has to find a self consistent solution for this set of equations. One possible approach would be to use a global numerical optimizer such as a basin-hopping algorithm to find a solution in the 7-dimensional parameter space spanned by $\{\phi, h, \langle R\rangle, q_n, q_R, \nu, \chi\}$. This requires non-convex optimization in high dimension, which is not guaranteed to work. By some additional manipulation we were able to reduce it into a one dimensional non-convex optimization over the variable Δ_g, as we now show.

Simplifying the expressions for the susceptibilities results with the third order polynomial for ν where the only unknown is ϕ.

$$\omega^2\gamma\sigma_d^2\sigma_c^2\nu^3-\omega^2\gamma\sigma_d^2\nu^2-\left(\frac{M}{S}\sigma_c^2-\phi_S\sigma_c^2\right)\nu-\phi_S=0$$

Note that ϕ only depends on Δ_g, therefore one can span a grid of values for Δ_g and assigning the roots the above polynomial for each $\nu_i\left(\Delta_g\right)$ where $i=1,2,3$. Plugging back into the expression for resources susceptibility leads to $\chi_i\left(\Delta_g\right)$.

Now, using the relations $\sigma_g h=-\frac{1}{\nu}w_0\left(\Delta_g\right)/w_1\left(\Delta_g\right)$ and $\Delta_g=\frac{g}{\sigma_g}$ yields

$$\left(\frac{M}{S}\mu_c\chi K-m\right)h-\frac{M}{S}\mu_c^2\chi-\omega\mu_d=-\Delta_g\frac{1}{\nu}\frac{w_0\left(\Delta_g\right)}{w_1\left(\Delta_g\right)}$$

solving this for $h\left(\Delta_g\right)$ leads to

$$h_i\left(\Delta_g\right)=\frac{\frac{M}{S}\mu_c^2\chi_i\left(\Delta_g\right)+\omega\mu_d-\frac{\Delta_g}{\nu_i\left(\Delta_g\right)}\frac{w_0\left(\Delta_g\right)}{w_1\left(\Delta_g\right)}}{\frac{M}{S}\mu_c K\chi_i\left(\Delta_g\right)-m}$$

Rewriting the expression for Δ_g with the new variables h, q_n

$$g=\frac{1}{h}\left[\frac{M}{S}\mu_c\chi\left(Kh-\mu_c\right)-\omega\mu_d-mh\right]$$

$$\sigma_g=\frac{1}{h}\left\{\begin{array}{c}\frac{M}{S}\chi^2\sigma_c^2\left[\sigma_K^2h^2+\sigma_c^2q_n+\left(Kh-\mu_c\right)^2\right]\\+\sigma_m^2h^2+\omega^2\sigma_d^2q_n\end{array}\right\}^{1/2}$$

$$\hat{\Delta}_g=\frac{\frac{M}{S}\mu_c\chi\left(Kh-\mu_c\right)-\omega\mu_d-mh}{\left\{\begin{array}{c}\frac{M}{S}\chi^2\sigma_c^2\left[\sigma_K^2h^2+\sigma_c^2q_n+\left(Kh-\mu_c\right)^2\right]\\+\sigma_m^2h^2+\omega^2\sigma_d^2q_n\end{array}\right\}^{1/2}}$$

Finally, find values of Δ_g and $i=1,2,3$ where $\hat{\Delta}_g\left[h_i\left(\Delta_g\right)\right]=\Delta_g$. By plugging in the self consistent values Δ_g, h, ν, χ into Eq. (C1) one can find $\{\phi, \langle N\rangle, \langle R\rangle, q_N, q_R\}$ for the set of control parameters at hand.

Appendix D: Random Matrix Theory

Given the values of control parameters as described in Appendix A, the diversity $\phi=S^*/S$ for the perturbed MCRM (Sec. II B) can be found as described in Appendix C. Here we define the random matrix ensemble corresponding to the reduced interaction matrix for given control parameters and diversity values. The main result of this appendix is the minimal eigenvalue real part of the ensemble Eq. (D1) in Appendix D 3. This in turn is used to distinguish between the stable and non-equilibrium phases in Fig. 3.

1. Random matrix theory and free probability

The linear stability of a fixed point is determined by the sign of the minimal eigenvalue of its interaction matrix. For randomly sampled interaction matrices, the problem of determining the sign of the minimal eigenvalue can be addressed with random matrix theory (RMT). A random matrix is a matrix whose elements are drawn from probability distribution, known as an ensemble. One of the main uses of RMT is to determine what the spectrum of a typical matrix drawn from such ensemble would look like, and in particular its minimal eigenvalue. Below we describe the key steps taken to find the minimal eigenvalue of the particular ensemble at hand. For a detailed review of these techniques see [7].

A central object in RMT is the Green function of an ensemble, also known as a Resolvent or Stieltjes transform. For an $N \times N$ random matrix H, the Green function is defined as

$$G_N(z) = \frac{1}{N} Tr\left([z\mathbb{I} - H]^{-1}\right) = \frac{1}{N}\sum_{i=1}^{N}\frac{1}{z - x_i}$$

where $x_1, \ldots x_N$ are the eigenvalues of H. Since H is a random matrix, $G_N(z)$ is a random complex function with poles at locations x_i. There are several methods for deriving the green function for a given ensemble, for details see [7]. Averaging over H and taking the thermodynamic limit $(N \to \infty)$,

$$G(z) = \lim_{N\to\infty}\langle G_N(z)\rangle = \int dx \frac{\rho(x)}{z - x} \ .$$

At the thermodynamic limit the set of eigenvalues $x_1, \ldots x_N$ becomes the eigenvalue density $\rho(x)$ for the ensemble. Using the Sokhotski-Plemelj formula one can extract the eigenvalue density $\rho(x)$ from the green function $G(z)$ as follows

$$\rho(x) = \frac{1}{\pi}\lim_{\epsilon\to 0^+} Im\left[G(x - i\epsilon)\right]$$

In this work, we want to calculate properties of sums of random matrices (the sum of the MCRM interactions and direct interactions). In general, random matrices do not commute, and the spectrum of the sum matrix isn't simply the sum of the spectra. Therefore it is hard to calculate the spectrum of random matrices sum even given access to the Green functions of the ensembles. Free probability is a tool generalizing the notion of random variable independence to the field of random matrices. Analogous to statistical independence for random variables, two random matrix ensembles may exhibit the 'freeness' property, the precise definition can be found in [7].

Free probability provides us with a prescription for deriving the Green function of the ensemble sum given the Green function of the summed ensembles exhibiting the freeness property. This is analogous to the convolution law for random variable sum. It proceeds as follows. First, define the complex valued blue function to be the functional inverse of the green function

$$G(B(z)) = z$$

Now, given the blue function of the two ensembles $B_1(z), B_2(z)$, the blue function of the sum ensemble reads

$$B(z) = B_1(z) + B_2(z) - \frac{1}{z} \ .$$

Finally, to find the green function of the sum ensemble, invert the blue function above using the relation

$$B(G(z)) = z \ .$$

2. Wishart, GOE and Ginibre ensembles

The perturbed MCRM interaction matrix appearing in Sec. II C consists of a sum of two matrices:

1. Resource competition interaction matrix - Wishart ensemble

$$\alpha_{ij}^{(r)} = \sum_{\beta=1}^{M} c_{i\beta}c_{j\beta}, \quad c_{i\beta} \sim Norm\left(\frac{\mu_c}{S}, \frac{\sigma_c}{\sqrt{S}}\right)$$

with the blue function

$$B_W(z) = \sigma_c^2\frac{\kappa}{1 - \sigma_c^2 z} + \frac{1}{z}; \quad \kappa = \frac{M}{N} \ .$$

2. Direct competition interaction matrix - Ginibre ensemble

$$\alpha_{ij}^{(d)} \sim Norm\left(\frac{\mu_d}{S}, \frac{\sigma_d}{\sqrt{S}}\right) \ ; \ corr\left(\alpha_{ij}^{(d)}, \alpha_{ji}^{(d)}\right) = \gamma \ .$$

In general (for $\gamma \neq 1$) a matrix drawn from the Ginibre ensemble is not Hermitian and therefore has a complex valued spectrum. Non Hermitian ensembles call for a generalization of the Green function. Concretely, in these cases the Green function would be a Quaternionic valued function leading to much more complicated calculations. Luckily, the Ginibre ensemble can be assembled as the sum of two independently distributed matrices from the Gaussian orthogonal ensemble (GOE) with complex prefactors [8]. This representation of the Ginibre ensemble allows for great simplification following method by [9]. Given two matrices H, H', with elements drawn independently from $H_{ij} \sim Norm\left(0, \sigma^2/N\right)$ and symmetrize $\left(H + H^T\right)/2$. The Ginibre matrix $\alpha^{(d)}$ can be written as

$$\alpha^{(d)} = c_2 H + i c_3 H'$$
$$c_2 = \sigma_d \omega\sqrt{1 + \gamma}$$
$$c_3 = \sigma_d \omega\sqrt{1 - \gamma}$$

where ω is the aggression factor maintaining a constant direct perturbation strength as described in II B. The blue function of the GOE with real value prefactor c is given by

$$B_{c\cdot GOE}(z) = \frac{1}{2}c^2 z + \frac{1}{z}$$

Finally, the ensemble for the perturbed interaction matrix $\alpha = \alpha^{(r)} + \omega \cdot \alpha^{(d)}$ can be written as

$$\begin{aligned}\alpha &= c_1 W + c_2 H + i c_3 H' \\ c_1 &= \sigma_c^2 \\ c_2 &= \sigma_d \omega \sqrt{1+\gamma} \\ c_3 &= \sigma_d \omega \sqrt{1-\gamma}\end{aligned}$$

with the blue functions for the real and imaginary parts

$$B_{\Re\alpha}(z) = B_{c_1 W} + B_{c_2\cdot GOE} - \frac{1}{z} = c_1 \frac{\kappa}{1 - c_1 z} + \frac{1}{2}c_2^2 z + \frac{1}{z}$$

$$B_{\Im\alpha}(z) = \frac{1}{2}c_3^2 z + \frac{1}{z}$$

3. Calculating the minimal eigenvalue of the matrix sum

In this section we derive the minimal eigenvalue real part for the ensemble describing the reduced interaction matrix of the perturbed MCRM in Sec. II B. This is the main result of this appendix, then being utilized to find the phase diagram in Fig. 3.

In this section we follow the method by [9] to find the spectrum of the sum of Hermitian random matrices with imagery prefactors. Using this method one can derive the spectrum of a non-Hermitian random matrix $H_1 + iH_2$ comprised of two Hermitian matrices H_1, H_2, without having to go through cumbersome calculations green functions quaternionic.

Still, it is hard to find the entire spectrum of this ensemble for α. We simplify the problem further, trying to find just the minimal real part of the complex spectrum, given as a particular case of the equations for spectrum support contour on the complex plane.

$$g \equiv a + ib \; ; \qquad g^I = \alpha + i\beta$$

By [9], Eq. (77),

$$B_H(g) = \frac{c_1\kappa}{1 - c_1 g} + \frac{1}{2}c_2^2 g + \frac{1}{g}$$

$$gB_H(g) = \frac{1}{2}c_2^2\left(a^2 - b^2 + 2iab\right) + c_1\kappa\frac{(1-c_1 a)a - c_1 b^2 + ib}{(1-c_1 a)^2 + c_1^2 b^2} + 1$$

$$\frac{gB_H(g) - \bar{g}B_H(\bar{g})}{g - \bar{g}} = c_2^2 a + c_1\kappa\frac{1}{(1-c_1 a)^2 + c_1^2 b^2}$$

$$\Downarrow$$

$$x = c_2^2 a + \frac{c_1\kappa}{(1-c_1 a)^2 + c_1^2 b^2}$$

By [9], Eq. (78),

$$B_{H'}\left(g^I\right) = \frac{1}{2}c_3^2 g^I + \frac{1}{g^I}$$

$$g^I B_{H'}\left(g^I\right) = \frac{1}{2}c_3^2\left(g^I\right)^2 + 1 = \frac{1}{2}c_3^2\left(\alpha^2 - \beta^2 + 2i\alpha\beta\right) + 1$$

$$g^I B_{H'}\left(g^I\right) - \bar{g}^I B_{H'}\left(\bar{g}^I\right) = 2ic_3^2\alpha\beta$$

$$g^I B_{H'}\left(g^I\right) - \bar{g}^I B_{H'}\left(\bar{g}^I\right) = y\left(g^I - \bar{g}^I\right)$$

$$\Downarrow$$

$$y = \frac{\alpha}{c_3^2}$$

And from [9], Eq. (74),

$$\frac{B_H(g) - B_H(\bar{g})}{g - \bar{g}} + \frac{B_{H'}\left(g^I\right) - B_{H'}\left(\bar{g}^I\right)}{g^I - \bar{g}^I} + \frac{1}{g\bar{g}} = 0$$

$$\begin{aligned}B_H(g) &= \frac{c_1\kappa}{1 - c_1 g} + \frac{1}{2}c_2^2 g + \frac{1}{g} \\ &= c_1\kappa\frac{1 - c_1 a + c_1 ib}{(1-c_1 a)^2 + c_1^2 b^2} + \frac{1}{2}c_2^2 g + \frac{\bar{g}}{g\bar{g}}\end{aligned}$$

$$\frac{B_H(g) - B_H(\bar{g})}{g - \bar{g}} = c_1\kappa\frac{c_1}{(1-c_1 a)^2 + c_1^2 b^2} + \frac{1}{2}c_2^2 - \frac{1}{a^2 + b^2}$$

Using [9], Eq. (63) we have $g\bar{g} = g^I \bar{g}^I$

$$\frac{B_{H'}\left(g^I\right) - B_{H'}\left(\bar{g}^I\right)}{g^I - \bar{g}^I} = \frac{1}{2}c_3^2 - \frac{1}{\alpha^2 + \beta^2} = \frac{1}{2}c_3^2 - \frac{1}{a^2 + b^2}$$

$$\Downarrow$$

$$\frac{c_1^2\kappa}{(1-c_1 a)^2 + c_1^2 b^2} + \frac{1}{2}\left(c_2^2 + c_3^2\right) - \frac{1}{a^2 + b^2} = 0$$

Combining [9], Eqs. (63,74,77) we get the set of coupled equations

$$\begin{cases} x = c_2^2 a + \frac{c_1 \kappa}{(1-c_1 a)^2 + c_1^2 b^2} \\ \frac{c_1^2 \kappa}{(1-c_1 a)^2 + c_1^2 b^2} + \frac{1}{2}\left(c_2^2 + c_3^2\right) - \frac{1}{a^2+b^2} = 0 \end{cases}$$

According to [9], Eq. (93) the spectrum contour equation is given by $(g+\bar{g})^2 + \left(g^I + \bar{g}^I\right)^2 = 4g\bar{g}$. Now, focusing on the real part of the contour, given by $y = 0$ combined with [9], Eq. (78) leads to $\alpha = 0$. Therefore the contour equation reduce to

$$(g+\bar{g})^2 = 4g\bar{g} \Rightarrow 4a^2 = 4\left(a^2 + b^2\right) \Rightarrow b^2 = 0$$

Plugging that back to the set of coupled equations above, yields the polynomial equation for a

$$\begin{aligned} x &= c_2^2 a + \frac{c_1 \kappa}{(1-c_1 a)^2} \qquad \text{(D1)} \\ 0 &= c_1^2\left(c_2^2 + c_3^2\right) a^4 - 2c_1\left(c_2^2 + c_3^2\right) a^3 + \\ &+ \left(2(\kappa - 1) c_1^2 + c_2^2 + c_3^2\right) a^2 + 4c_1 a - 2 \end{aligned}$$

Substituting back the real a roots into Eq. (D1) to get the minimal and maximal eigenvalue real parts of the ensemble. By doing so determining the linear stability of the perturbed MCRM model at Eq. (2).

Appendix E: Species abundance distribution

At fixed points of resource-consumer models, species diversity is limited by the number of limiting resources ($S^* \leq M$), according to the competitive exclusion principle [10]. In contrast, non-equilibrium states are not bound by the exclusion principle and can exceed this limit, i.e. $M < S^*$. In this section we show that for the system described in Eq. (3) this is indeed the case, even if the migration is very small (in the $\eta \to 0$ limit).

As discussed in Sec. II E, simulations with migration η, show a typical species abundance probability distribution, see Fig. 5(A), with a power law as the abundance distribution between an upper value N_u, and lower value determined by the migration floor η. We write this probability distribution as

$$P(N) = \begin{cases} P_{high}(N) & N_u < N \\ cN^{-(\nu+1)} & b\eta < N < N_u \\ P_{low}(N) & \eta \lesssim N < b\eta \end{cases}$$

The power law behavior is parameterized by c, ν that may dependent on the migration η. The lower part $P_{low}(N)$ is the abundance distribution for species that are maintained thanks to migration. We define this region to go up to $b\eta$ with a (somewhat arbitrary) constant value b.

Our interest is in when and how species diversity goes beyond the competitive exclusion bound in this probability distribution. Let us define C_{high}, N_{CE}, C_{CE} as

$$\int_{N_u}^{\infty} P(N)\, dN \equiv C_{high} \ ,$$

$$\int_{N_{CE}}^{\infty} P(N)\, dN = \frac{M}{S} \ ,$$

$$\int_{b\eta}^{N_{CE}} P(N)\, dN \equiv C_{CE} \ .$$

Note that if competitive exclusion holds and $\eta = 0$, there are no species in the range $0 < N < N_{CE}$. That is, species are either extinct and concentrated at $N = 0$ of $P(N)$, or have an abundance above N_{CE}. Therefore, to demonstrate that the species diversity can exceed the competitive exclusion limit in chaotic states we show that $0 < C_{CE}$ at the limit of vanishing migration.

For simplicity of the analysis we replace P_{low} by extending the power law and introducing a new lower cutoff at $a\eta$, to preserve the area under the curve of this lower region. We still treat abundances below $b\eta$ as species maintained solely by migration. The simplified probability distribution reads

$$P(N) = \begin{cases} P_{high}(N) & N_u < N \\ cN^{-(1+\nu)} & a\eta < N < N_u \end{cases} .$$

From normalization of $P(N)$ we solve for $\frac{c}{\nu}$ to find

$$1 - C_{up} = \int_{a\eta}^{N_u} cN^{-(1+\nu)} dN = -\frac{c}{\nu}\left[N_u^{-\nu} - (a\eta)^{-\nu}\right] \ ,$$

$$\frac{c}{\nu} = \frac{(1 - C_{up})}{(a\eta)^{-\nu} - N_u^{-\nu}} \ .$$

Next, we express N_{CE} as

$$\frac{M}{S} - C_{up} = \int_{N_{CE}}^{N_u} cN^{-(1+\nu)} dN = -\frac{c}{\nu}\left[N_u^{-\nu} - N_{CE}^{-\nu}\right]$$

$$N_{CE}^{-\nu} = (a\eta)^{-\nu}\left(\frac{\frac{M}{S} - C_{up}}{1 - C_{up}}\right) + N_u^{-\nu}\left(\frac{1 - \frac{M}{S}}{1 - C_{up}}\right)$$

Finally, C_{CE} takes the form

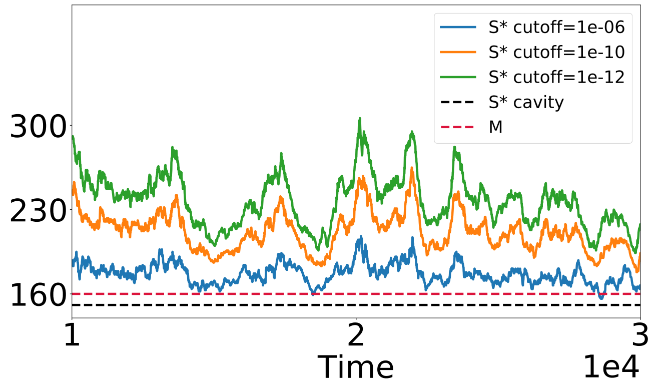

Figure A: Number of coexisting species S^*, as a function of time in the persistent dynamics phase. A species is counted in the standing diversity S^* if its abundance is above the level given in the legend. As can be seen, the number coexisting species exceeded the amount of resources for large range of abundance levels.

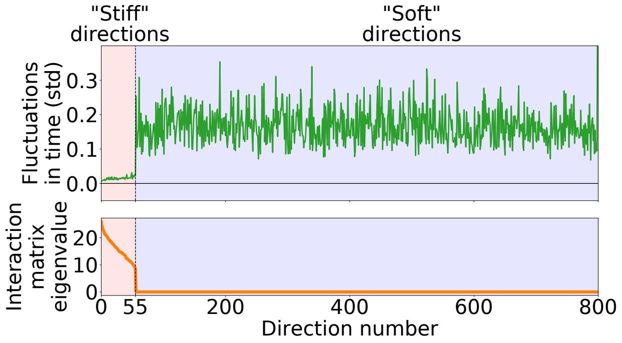

Figure B: (Top) Fluctuations over time along the eigenvector directions of the resource interaction matrix $\alpha^{(r)}$. (Bottom) Corresponding eigenvalues λ_i of the spectral decomposition of $\alpha^{(r)}$. There is a clear distinction between "Stiff" directions showing little fluctuations corresponding to finite positive eigenvalues, and "Soft", strongly fluctuating marginal directions with corresponding zero eigenvalues.

$$C_{CE}=\int_{b\eta}^{N_{CE}}\tilde{c}N^{-(1+\nu)}dN=-\frac{c}{\nu}\left[N_{CE}^{-\nu}-(b\eta)^{-\nu}\right]$$

$$C_{CE}=\frac{(b\eta)^{-\nu}-(a\eta)^{-\nu}}{(a\eta)^{-\nu}-N_u^{-\nu}}\left(1-C_{up}\right)+\left(1-\frac{M}{S}\right)$$

At the limit $\eta\rightarrow 0$

$$C_{CE}=\left(\frac{a}{b}\right)^{\nu}\left(1-C_{up}\right)+C_{up}-\frac{M}{S}$$

This equation expresses C_{CE} as a function of the parameters of the probability distribution (a,b,ν,C_{up}), and the number of species M and resources S. Simulations shows that $0\leq\nu\ll 1$ (possibly vanishing) at the limit $\eta\rightarrow 0$. Assuming that $M/S<1$ (there are more species in the pool than resources), we conclude that $0<C_{CE}$, hence non-equilibrium states of consumer-resource models can sustain diversity exceeding the competitive exclusion limit.

Finally, a note regarding the diversity, compared with the diversity as predicted by the cavity solution described in Appendix C. That solution is only exact when the system reaches a unique stable equilibrium. Elsewhere it is an approximation; from simulations of the first model variant in Sec. II B, we find that the cavity solution is lower than the one described in this Section, see Fig. A.

Appendix F: Stiff and soft fluctuation directions

It is interesting to see whether the fluctuations of the abundances are directly related to the marginal directions of the MCRM fixed points. To do that, we rotate the vector $\{N_i\}_{i=1}^{S}$ in a way that will separate the "stiff" degrees of freedom, lying in the non-marginal directions of a MCRM fixed point, and the "soft" degrees of freedom at the marginal dimensions. This is done by rotating with an orthogonal matrix O the abundance vector $\vec{N}(t)$ from a simulation of the perturbed MCRM in it's chaotic phase. The orthogonal matrix O is obtained from the spectral decomposition of the unperturbed interaction matrix $\alpha_{ij}^{(r)}=\sum_{k,l}O_{ik}D_{kl}O_{jl}$ where D is diagonal matrix. Note that since $\alpha^{(r)}$ is a symmetric positive-semi-definite matrix, its eigenvalues $\{\lambda_i\}_{i=1}^{S}$ are real valued and non-negative.

Denote by $y_i(t)=\sum_{j=1}^{S}O_{ji}N_i(t)$ the rotated degrees of freedom. These are a combination of species abundances at time t. Define the fluctuation over time in direction i to be $\mathrm{std}(y_i)\equiv\sqrt{\langle y_i(t)-\langle y_i(t)\rangle_t\rangle_t}$, where $\langle\ldots\rangle_t$ denotes time average over a $\Delta t=10^4$ interval . Plotting $\mathrm{std}(y_i)$ sorted by the eigenvalue λ_i, shows that the fluctuations in the "soft" directions (where $\lambda_i\doteq 0$) are consistently and significantly larger than in the "stiff" directions (where $\lambda_i>0$), see Fig. B.

Appendix G: Isolated systems (no migration)

Here we discuss cases where there is no migration from an external "mainland" pool of species. We consider both a single community, and a a meta-community, a setting in which multiple well-mixed communities are coupled by migration. We show that meta-communities can allow for persistent dynamics over long times, even in the absence of external migration, and for finite population sizes. In isolated well-mixed communities, simulations show that extinctions drive the system to a fixed point, with diversity a little below the competitive exclusion bound.

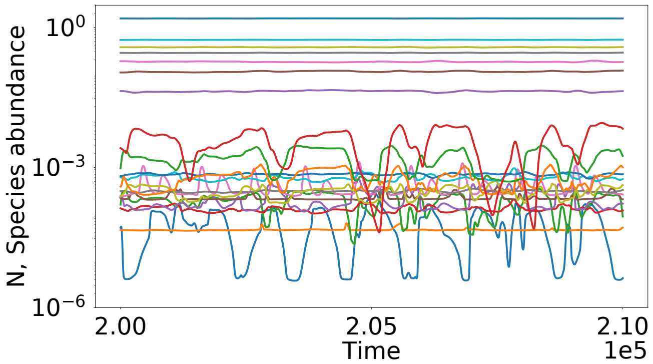

Figure C: Dynamics of a meta-community composed of 8 communities coupled by migration, at late times. Persistent abundance fluctuations are shown, which do not go below some value, showing that even finite populations can persist for very long times. 20 representative species are plotted.

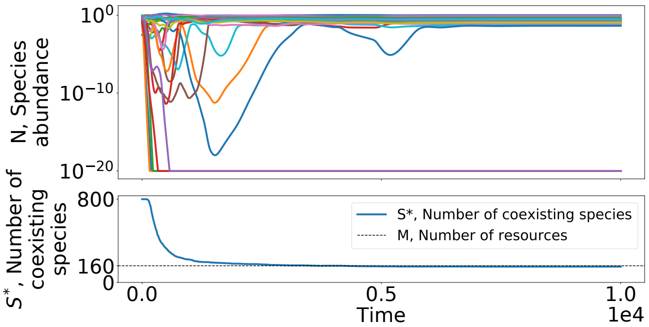

Figure D: Dynamics of a single community without external migration at the chaotic phase. Species abundances initially fluctuate, and some go extinct. A fixed point is reached once the diversity goes a little below the number of resources M.

The dynamics of the meta-community are a set of differential equations for $N_i^{(u)}$ describing the abundance of the i-th species in the u-th community,

$$\frac{dN_i^{(u)}}{dt} = ... + \sum_v D_i^{(u,v)} \left[N_i^{(v)} - N_i^{(u)} \right] \ ,$$

where the "..." refers to the terms in the RHS of Eq. (2) applied to $N_i^{(u)}$, with $\eta_i = 0$. A species is considered extinct and removed from the system when its abundance $N_i^{(u)}$ goes below some cut-off N_c in all communities u, corresponding to the inverse of the population size.

Fig. C shows the dynamics at late times of a few representative abundances $N_i^{(u)}$, showing persistent fluctuations in a meta-community comprised of 8 communities with $S = 400$ species and $M = 80$ resources. The model in each patch corresponds to that in Sec. II B. The resource interaction matrix $\alpha^{(r)}$ has $\mu_c = 30$ and $\sigma_c = 6$. The matrix α is very similar but not identical between the different communities, with correlation $\rho = 0.997$ between the $\alpha_{ij} = \alpha_{ij}^{(r)} + \omega\alpha_{ij}^{(d)}$ for the same i, j in different communities. Direct interaction matrix $\alpha^{(d)}$ is drawn independently for each community with $\mu_d = 10$, $\sigma_d = 20$ and $\gamma = 0$. As in the main text, ω is determined to satisfy perturbation strength of 0.05. Coupling between communities set to be $D = 10^{-4}$. Cutoff abundance is taken to be $N_c = 10^{-20}$.

A simulation of a single, well-mixed community is shown in Fig. D, along with the diversity as a function of time. The diversity drops, until the system reaches a fixed point with S^* a little below M, almost saturating the competitive exclusion bound. Simulation parameters are specified in Tab. I and are similar to that in Fig. 1(C) but with $\eta = 0$.

Appendix H: Symmetric additional interactions

Here we consider a setting similar to that in Sec. II B, where additional interactions $\alpha_{ij}^{(d)}$ are added to the ground model. The difference is that here they are taken to be symmetric, $\alpha_{ij}^{(d)} = \alpha_{ji}^{(d)}$. This difference is important, since in this case, the entire interaction matrix α is symmetric. This means that the dynamics admit a Lyapunov function, and always reach a fixed point. A similar situation, with symmetric random Lotka-Volterra interactions (in this work's terminology, $\alpha = \alpha^{(d)}$) has been studied in [11]. There, a fixed point phase was found. Beyond it lies a *critical* phase, characterized by many alternative equilibria, all of them close to marginal stability, namely such that the minimal eigenvalue $\lambda_{min} \to 0$ as $S \to \infty$. Specifically, it was found that $\lambda_{min} \propto S^{-2/3}$.

Here we find precisely the same phenomenology, with a fixed point phase. Beyond it simulations show that the system possesses multiple alternative equilibria. Furthermore, the minimal eigenvalue was measured for multiple values of σ_c and S, and averaged over many runs. For each value of σ_c it was fit to $\lambda_{min}(S) = a \cdot S^b + \lambda_\infty$, where a, b, λ_∞ depend on σ_c, see Fig. E. The results for b, λ_∞ are shown in Fig. F. We find that beyond the unique fixed point phase the results are very different from the simple cavity solution for this case, and consistent with $\lambda_\infty = 0$ and $b = -2/3$ which was predicted for the random Lotka-Volterra setting.

Appendix I: A model with explicit resources and no abundance divergence

In this Appendix we look at a model which addresses certain features of the model that we use in Sections II B,II C, changing them to more biologically plausible alternatives. We show that all the qualitative features discussed in Sections II B,II C are also present in this more biologically realistic model.

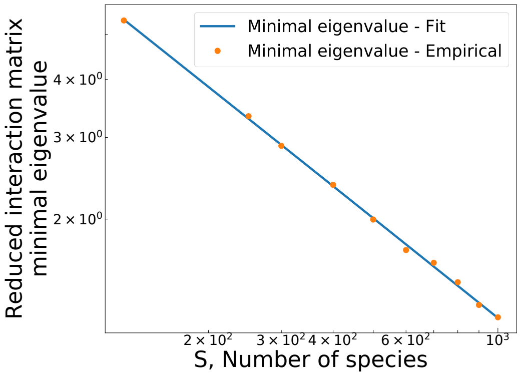

Figure E: Fitting the minimal eigenvalue of the reduced interaction matrix to $\lambda_{min}(S) = a \cdot S^b + \lambda_\infty$. Parameters as in Fig. F, with $\sigma_c = 23$.

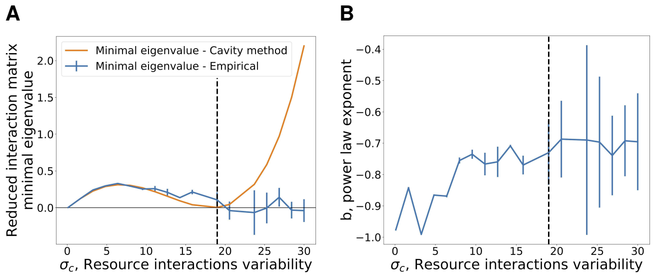

Figure F: Minimal eigenvalue of the reduced interaction matrix with symmetric direct interactions perturbation ($\gamma = 1$). Minimal eigenvalue at $S \to \infty$ is obtained from a fit to $\lambda_{min}(S) = a \cdot S^b + \lambda_\infty$, see Fig. E. Shown are (A) The minimal eigenvalue λ_∞, and (B) the power law exponent b.

More specifically, in the main text we use Eq. (2) which is obtained from Eq. (1) by substituting the resource values. As explained there, this means that resource values might go negative. In this Appendix we therefore start from the original equation (1) which includes explicit resource values, and add a small migration to the resources, to prevent them from going negative.

Secondly, in the model used in the main text, the abundances diverge for some parameter regime. Of course, abundances cannot diverge, and this means that at higher values they must encounter stronger limitations on growth. We model this by an additional term, $dN_i/dt = N_i\left[.. - \kappa N_i^2\right]$. This happens away from the transition from equilibria to persistent dynamics, and indeed we show that it has a negligible effect on the behavior away from where abundances diverge. Together, the equations read

$$\begin{cases} \frac{dN_i}{dt} = N_i\left[\sum_\beta c_{i\beta}R_\beta - m_i - \omega\sum_j \alpha_{ij}^{(d)}N_j - \kappa N_i^2\right] + \eta_i \\ \frac{dR_\beta}{dt} = R_\beta\left[K_\beta - R_\beta - \sum_j c_{j\beta}N_j\right] + \eta_\beta \end{cases} \tag{I1}$$

Finally, because the consumer preferences $c_{i\beta}$ are drawn from a Gaussian distribution, a small fraction of them might be negative, $c_{i\beta} < 0$, which is hard to interpret as resource consumption. We therefore set any negative $c_{i\beta}$ to zero.

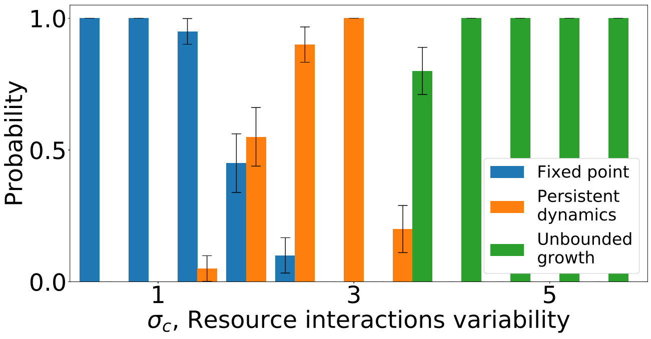

Figure G: Fraction of simulations that reach equilibria, persistent dynamics or diverge. The dynamics follow Eq. (I1) with interactions drawn for different values of σ_c, the variability in consumer preferences, and $\kappa = 0$. The probability is estimated from 20 simulations for each value of σ_c. Errorbars give the standard error of the estimate. Other control parameters used in this simulation can be found in Table (I).

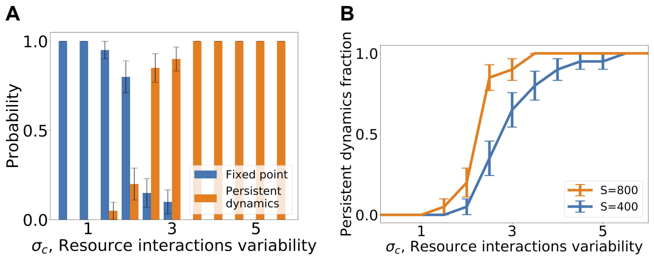

Figure H: (A) Fraction of simulations that reach equilibria, persistent dynamics or diverge. Model as in Fig. G, but with stronger limitation on growth of abundances, $\kappa = 10^{-2}$. Species abundances remain finite for all values of σ_c. (B) The transition between fixed point and persistent dynamics becomes sharper as system size increases. Other control parameters used in this simulation can be found in Table (I).

As in the main text, as parameters are changed we see a transition from equilibrium to persistent dynamics, see Fig. G. This is with $\kappa = 0$, so abundances are able to diverge, as they do in some parameter regimes. When growth rate is more strongly limited by setting $\kappa > 0$, see Fig. H(A), the unbounded growth is removed for all parameter values, with little effect on the transition from the equilibria to persistent dynamics regimes. As the number of species S is increased, the transition becomes sharper, see Fig. H(B), just like in the main text Fig. 3(C).

[1] H. Rieger. Solvable model of a complex ecosystem with randomly interacting species. *Journal of Physics A: Mathematical and General*, 22(17):3447, 1989.

[2] Manfred Opper and Sigurd Diederich. Phase transition and 1/f noise in a game dynamical model. *Physical review letters*, 69(10):1616, 1992.

[3] Marc Mézard, Giorgio Parisi, and M. A. Virasoro. SK model: The replica solution without replicas. *EPL (Europhysics Letters)*, 1(2):77, 1986.

[4] A. Crisanti, H. Horner, and H.-J. Sommers. The sphericalp-spin interaction spin-glass model. *Zeitschrift für Physik B Condensed Matter*, 92(2):257–271, 1993.

[5] Guy Bunin. Ecological communities with Lotka-Volterra dynamics. *Physical Review E*, 95(4), April 2017.

[6] Madhu Advani, Guy Bunin, and Pankaj Mehta. Statistical physics of community ecology: A cavity solution to MacArthur's consumer resource model. *Journal of Statistical Mechanics: Theory and Experiment*, 2018(3):033406, March 2018.

[7] Giacomo Livan, Marcel Novaes, and Pierpaolo Vivo. Introduction to Random Matrices - Theory and Practice. *arXiv:1712.07903 [cond-mat, physics:math-ph]*, 26, 2018.

[8] Zdzislaw Burda and Artur Swiech. Quaternionic R transform and non-Hermitian random matrices. *Physical Review E*, 92(5):052111, 2015.

[9] Andrzej Jarosz and Maciej A. Nowak. A Novel Approach to Non-Hermitian Random Matrix Models. *arXiv:math-ph/0402057*, February 2004.

[10] Richard McGehee and Robert A. Armstrong. Some mathematical problems concerning the ecological principle of competitive exclusion. *Journal of Differential Equations*, 23(1):30–52, 1977.

[11] Giulio Biroli, Guy Bunin, and Chiara Cammarota. Marginally stable equilibria in critical ecosystems. *New Journal of Physics*, 20(8):083051, August 2018.
